# Network analysis indicating the pharmacological mechanism of Yunpi-Qufeng-Chushi-prescription in prophylactic treatment of rheumatoid arthritis

**DOI:** 10.1186/s12906-021-03311-4

**Published:** 2021-05-15

**Authors:** Lin Li, Donghai Zhou, Qiuping Liu, Dianming Li, Qiao Wang, Xiaowei Shi, Chengping Wen, Lin Huang

**Affiliations:** 1 School of Basic Medical Sciences, Zhejiang Chinese Medical University, 548 Binwen Road, Hangzhou, 310000 Zhejiang China; 2 The Second Affiliated Hospital of Zhejiang Chinese Medical University, Hangzhou, 310005 China

## Abstract

**Background:**

Rheumatoid arthritis (RA), is an autoimmune inflammatory disease with increasing global morbidity and high disability. Early treatment is an effective intervention to slow down joint deformation. However, as for early RA and pre-RA patients, it sometimes takes a long time to make a definite diagnosis and few guidelines have made suggestion for these suspected or early phrase individuals. Yunpi-Qufeng-Chushi-Prescription (YQCP) is an optimization of the traditional formula, Cangzhu Fangfeng Tang which is effective for arthromyodynia management.

**Methods:**

In this study, LC-MS identify the main component of YQCP. Ingredients of the 11 herbs were collected from Traditional Chinese Medicine Integrated Database (TCMID). Targets of these ingredients were collected from two source, TCMID and PharmMapper. Microarray of 20 early untreated RA patients and corresponding health control were download from NCBI Gene Expression Omnibus (GEO) database to defined the differential expressed genes. Gene ontology analysis and KEGG enrichment analysis were carried out for the YQCP. Protein-protein interactions (PPIs) networks were constructed to identify the hub targets. At last, molecular docking (MD) were conducted to further verified the the possibility of YQCP for RA therapy.

**Result:**

The study indicated that by acting on hub targets such as C3, EGFR, SRC and MMP9, YQCP may influence the mature of B cells and inhibit B cell-related IgG production, regulate oxidative stress and modulate activity of several enzymes including peroxidase and metallopeptidase to delay the occurrence and progress of RA and benefit the pre-RA or early RA patients.

**Conclusion:**

YQCP is a potential effective therapy for prophylactic treatment of RA.

**Supplementary Information:**

The online version contains supplementary material available at 10.1186/s12906-021-03311-4.

## Introduction

Rheumatoid arthritis (RA), is an autoimmune inflammatory disease characterized by chronic, symmetrical arthritis and extraarticular lesions. The global incidence of RA is approximately 0.24% and RA ranks within the top 50 diseases that contribute to global disability [1]. Inflammation often occurs in facet joints of the hands, wrists and feet which would have an impact on normal life. RA is a multifactorial chronic disease and the precise aetiology is remain elusive. It is reported that heritability of RA was ranging from 15 to 60%, indicating genetic factor as one of the pathogenic factors [2]. In addition, a variety of environmental factors, immune cells and cytokines, such as smoking, T cells, B cells, TLRs (Toll-like receptor) and virus are involved in the pathological process [3–5].

There is a prolonged phrase in onset latency of RA when serum antibody was identified in the absence of arthralgia or synovitis. It is generally considered that early identification and treatment is of great importance in RA disease management. However, as for early RA patients, it sometimes takes months or years to make a definite diagnosis and few guidelines have made suggestion for these suspected individuals. Non-steroidal anti-inflammatory drugs help alleviate the pain but cannot delay the progression of joint destruction while disease-modifying anti-rheumatic drugs slow down joint deformity but only suggested to be applied after clinical diagnosis. Therefore, it is an urgent target to put forward medical advice for early RA patients.

Traditional Chinese Medicine (TCM), especially herbal medicine has long been used in inflammation disease including arthralgia. One of the prescriptions is Cangzhu Fangfeng Prescription which was recorded in “*Su Wen Bing Ji Qi Yi Bao Ming Ji*”. The TCM rheumatologist Professor Chengping Wen inherited the academic thoughts of the formula and continuous optimized it in clinic, finally coming up wtih Yunpi-Qufeng-Chushi prescription (YQCP). YQCP is consist of 9 basal herbs (Table 1), i.e. cang zhu (*Atractylodes lancea*), fang feng (*Saposhnikovia divaricata*), qing feng teng (*Sinomenium acutum*), jin yin hua (*Lonicera japonica*), hu zhang (*Polygonum cuspidatum*), yi yi ren (*Coix lacryma- jobi var. ma - yuen*), zhi gan cao (*Radix Glycyrrhizae Preparata*), tu fu ling (*Smilax glabra*), xu chang qing (*Cynanchum paniculatum*) and two alternative herbs designed for relieving severe joint pain, i.e. kun ming shan hai tang (*Tripterygium hypoglaucum*) and tu si zi (*Semen Cuscutae*). Some of these herbs have been demonstrated to prevent the aggravation of RA. For instance, triptolide, an extract of kun ming shan hai tang, suppresses human synoviocyte cells mobility and promote osteoclast apoptosis [6, 7]. Our research team have demonstrated that preventive treatment of YQCP can effectively delay the occurrence RA [8]. YQCP is an effective complementary alternative therapy for suspected RA patients who is lack of treatment guideline.

**Table 1:** Full scientific species names of herbs of Yunpi-Qufeng-Chushi-Prescription

Pin Yin	Latin Name
Cang Zhu	Atractylodes lancea
fang feng	Saposhnikovia divaricata
qing feng teng	Sinomenium acutum
jin yin hua	*Lonicera japonica*
hu zhang	*Polygonum cuspidatum*
yi yi ren	*Coix lacryma*- jobi var. ma - yuen
Xu Chang Qing	Cynanchum paniculatum
Zhi Gan Cao	Radix Glycyrrhizae Preparata
Tu Fu Ling	Smilax glabra
Kun Ming Shan Hai Tang	Tripterygium hypoglaucum
Tu Si zi	Semen Cuseutae

Because of the multi-ingredients and multi-targets hallmark, it is complex to figure out the underlying mechanism of TCM prescriptions. However, network pharmacology exhibits its superiority in addressing this issue and the result always offers effective advice for further experimental verification [9]. In this study, network pharmacology method was applied to unveil the potential molecular mechanism of YQCP and offers effective advice for conducting further research.

## Materials and methods

### Sample Preparation and UPLC-MS conditions

YQCP granules were produced by China Resources Sanjiu Pharmaceutical Factory. YQCP granules were ground into powder. 0.5 g powder were weighed for further testing. The powder was dissolved with 5mL 80% methanol and sonicated for 90 min at 35 kHz and 25 °C. After centrifugation at 3500 rpm for 10 min, 1 mL supernatant was taken which was then filtered with 0.22 μm filter and transferred to a 1.5 mL sample vial. The data of UPLC was acquired on Waters UPLC system (Waters UPLC I-CLASS and SYNAPT G2-Si) equipped with a C18 column (Inertsil ODS-2.1x100 mm, 1.6 μm). Solvent A was acetonitrile, and solvent B comprised 0.1% formic acid in water. 5 μL injection were eluted at 30 °C and a flow rate of 0.3 mL/min. using the following gradient program: 0-5% (0–2 min) solvent A, 5–100% solvent A (2–32 min). The mass spectrometer was operated in positive ion scanning modes with a capillary voltage of 2.5-3.0 kV.

### Dataset

Ingredients of the 11 herbs were collected from Traditional Chinese Medicine Integrated Database (TCMID) [10]. Targets of these ingredients were collected from two source, TCMID and PharmMapper [11]. TCMID and PharmMapper predicted the targets from two different approach, literature mining and molecular structure. Overlapped targets from these two databases were chosen as candidate targets. RA-related genes were get from human gene database GeneCards with the filter criteria of score > 5 [12].

### Microarray data processing of RA sample

Microarray of 20 early untreated RA patients and corresponding health control were download from NCBI Gene Expression Omnibus database (GSE45867). Expression values were normalized by MAS 5.0 function in R program. Differentially expressed genes (DEGs) were defined as genes whose fold change values were larger than 4 and adjust *P*-value less than 0.01. Annotations of microarray platforms (GPL570–55999) was used transformed the probe into gene official name, excluding probe with missing values for further analyses. One thousand five hundred seventy-nine genes were finally defined as DEGs. Hclust in R were utilized to compute the clustering distance of DEGs. The heatmap for the DEGs set was drawn using pheamap package in R.

### Network construction

Protein-protien interactions (PPIs) data was download from Human Protein Reference Database and Biogrid [13]. Herbal-ingredient-targets and PPI networks were visualized by cytoscape 3.6. MCODE plugin were used to filter out sub-cluster for the whole PPIs network.

### GO enrichment analysis

Gene Ontology (GO) analysis was carried out using Cluego plugin in cytoscape on immune process, molecular function and KEGG pathway for YQCP. GO Terms with *p*-value less than 0.01 were picked out.

### Molecular docking analysis

To analyze the feasibility of the main ingredients of YQCP in interaction with hub targets, we applied molecular docking analysis. The 2D structure of the 5 main ingredients were download from Pubchem database. The crystal structure of the 9 hub targets were got from PDB database. Selection principle of protein crystal structure includes *Homo sapiens* and good resolution of the 3D structure. H_2_O of the proteins were removed, and hydrogen atom were added in Pymol software. The docking was carried out using Autodocktools − 1.5.6. Binding energy of docking result was compared with the original ligand and binding energy less than − 5.0 kcal·mol^− 1^ were defined as dependable binding.

## Results

### LC-MS identify the main component

**Fig. 1:**
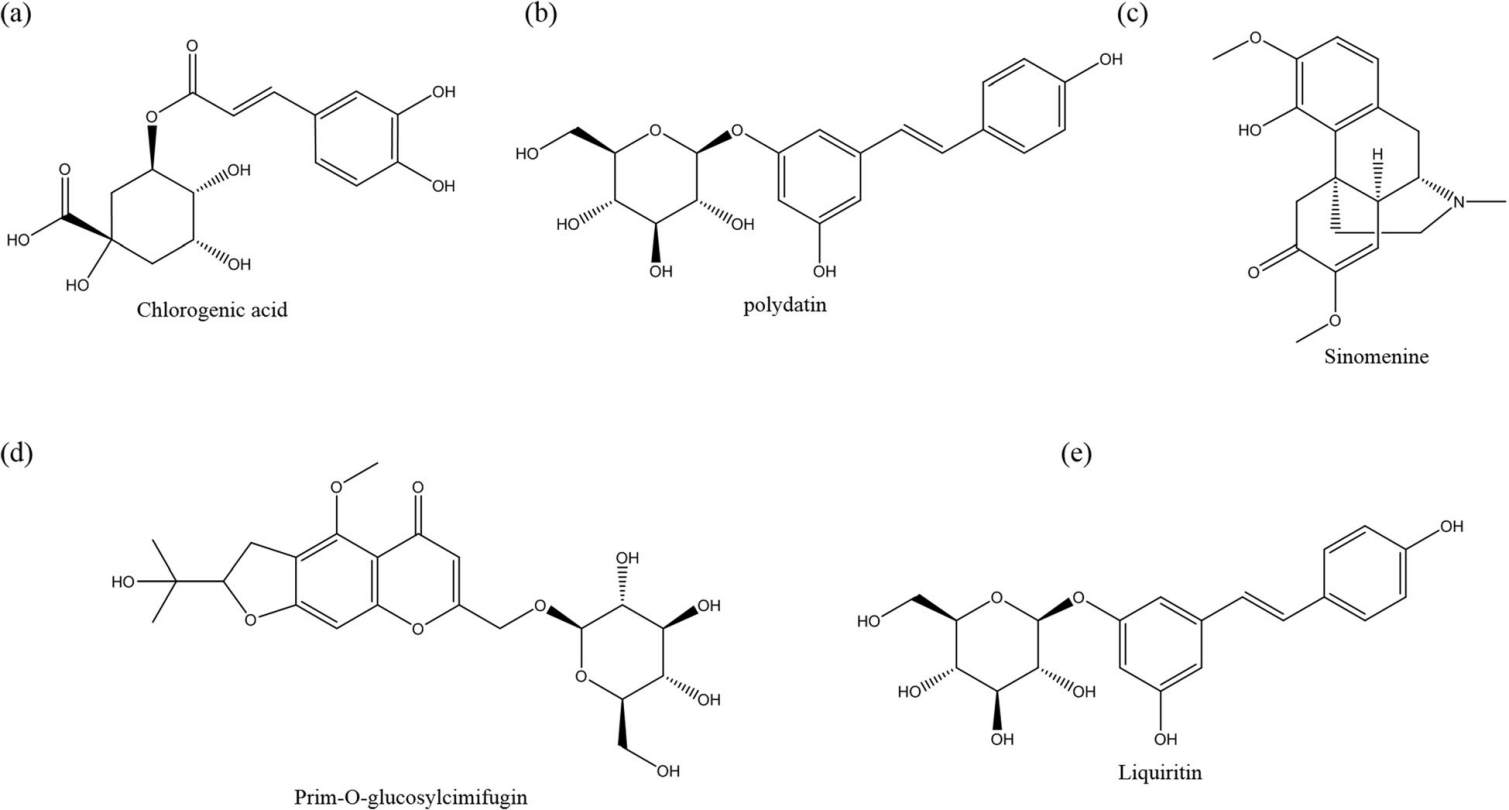
The molecular structure of main components. **a** chlorogenic acid, **b** polydatin, **c** sinomenine, **e** prim-o-glucosylcimifugin, **f** liquiritin

One hundred eleven kinds of component were identified by LC-MS (Table S1). According to literature research, we selected 5 main components, i.e. chlorogenic acid in *Lonicera japonica*, polydatin in *Rhizoma Polygoni Cuspidati*, prim-O-glucosylcimifugin in *Saposhnidoviae Radix*, sinomenine in *Caulis sinomenii* and liquiritin in *prepared Liquorice root* to show the structure (Fig. 1, Figure S1)*.* Batch information of each herbs were supplied in Table S2.

### Ingredients and targets of YQCP

**Fig. 2:**
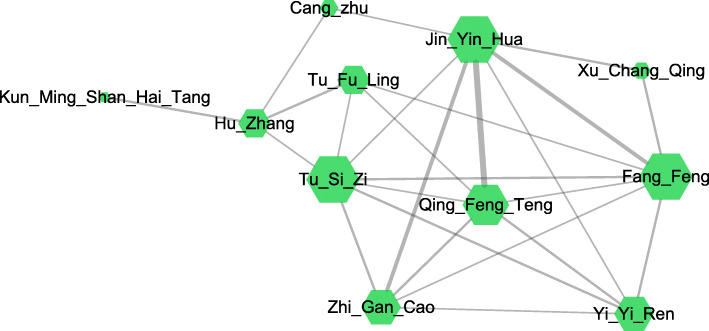
The relationships between the herbs sharing the same compounds

Six hundred ninety-nine ingredients from 11 herbs were collected from TCMID (Table S3). Twenty-three of them were shared between different herbs and the rest 676 were unique. As shown in Fig. 2, *Caulis sinomenii* and *Lonicera japonica* are the nearest ingredients. In the theory of TCM, these two herbs could clear away heat and toxic which is closed to anti-inflammatory painkillers in modern medicine. The overlapped ingredients include (+)-catechin, tryptophan and β-sitosterol. Therein, (+)-catechin could inhibit inflammatory milieu through IL-1β signaling [14] while tryptophan metabolism is involved in the initiation and propagation of synovitis [15]. β-sitosterol could influence macrophage polarization in RA mice and reduce inflammatory response [16]. The unique ingredients represent individuality of each herbs and are often the most effective ingredients. For instance, sinomenine, the typical component of *Caulis sinomenii* reduces cartilage destruction through inhibiting inflammatory signaling pathways like NF-κB [17].

Among the 699 ingredients, 46 of them has recorded targets in TCMID, providing 1822 predicted targets. Three hundred twenty-six of the ingredients were successfully matched to PharmMapper model, providing 3524 predicted targets. Four hundred thirteen targets are in common within the two database and defined as predicted formula targets, including C2, MMP2, MMP9, VEGFA (Table S4, supplementary data). Two hundred sixty-nine ingredients associated with these targets are defined as effective compound, including triptolide, sinomenine, atractylon, coixan A and so on.

### Functional analysis of YQCP predicted targets

**Fig. 3:**
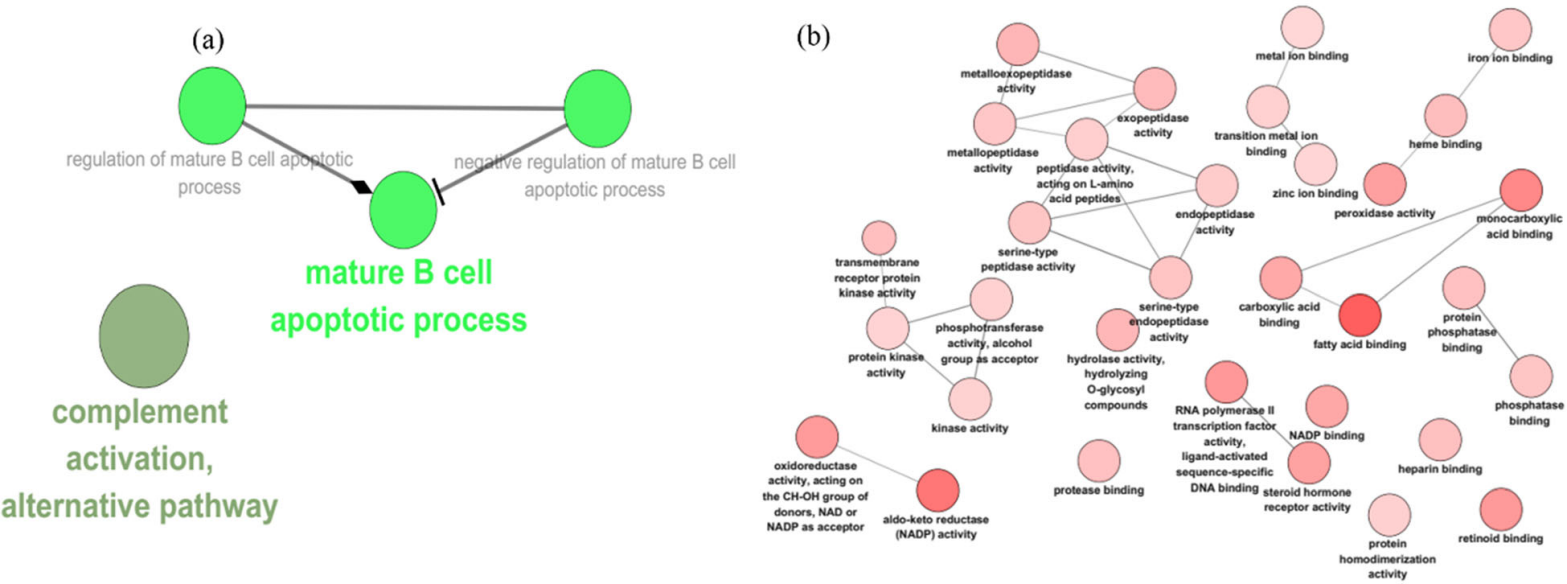
The gene ontology analysis of the YQCP predicted targets

GO analysis was carried for formula targets to predict the potential function of YQCP. On the aspect of immune system process, YQCP is mainly take part in the mature B cell apoptotic process and complement activation, alternative pathway on the GO level of 2 to 6 (*p*-value < 0.01) (Fig. 3a, Table S5). Higher percentage of peripheral B cells were found in RA patients as compared to healthy control [18]. Defects in the regulation of B cell apoptosis are required for the production of CCP [19]. Complement activation might be generated in at-risk individuals in local mucosal during preclinical [20]. In molecular function, enriched GO terms include metallopeptidase activity, transmembrane receptor protein kinase activity and peroxidase activity (Fig. 3b, Table S6). Excessive secretion of matrix metalloproteinases (MMPs) by fibroblast-like synoviocytes and peroxidase activity by neutrophils would lead to cartilage destruction [21, 22].

**Fig. 4:**
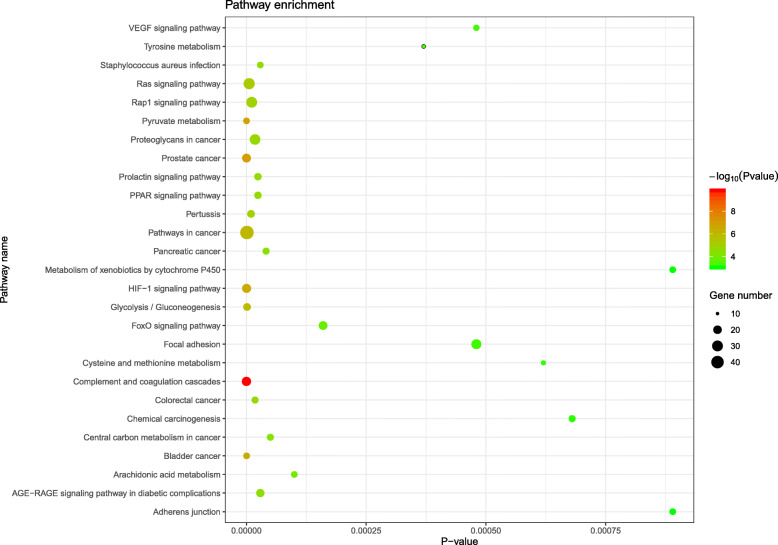
The KEGG enrichment analysis of the YQCP predicted targets

Moreover, formula targets enriched in 28 signaling pathways including arachidonic acid metabolism, adherens junction and complement and coagulation cascades (Fig. 4, Table S7). Cytokines released by B cells such as TNF-α and IL-1β stimulate the production of inflammatory factors of synovium and promote metallopeptidase activity [23]. Arachidonic acid metabolism pathway was demonstrated to be relate with mechanism of tocilizumab in dealing with early RA patients [24]. Adherens junction is indispensable components of the vessel wall that affect vascular permeability [25] while activation of complement and coagulation cascades modulates synovium and systemic inflammation [26]. The enrichment results suggested that function of YQCP is closely associated with RA therapy.

### PPI networks of finding the hub targets

Microarray of paired synovial biopsy samples obtained before and after treatment of 20 early RA patients were analyzed to define the DEGs relevant to treatment. Firstly, quality control by qc in R program confirm the quality of these chips. The raw data (CEL files) was then normalized by MAS 5.0. Then the contrast was made between RA samples before and after treatment using contrasts.fit and eBays. One thousand five hundred seventy-nine DEGs were filtered out with fold change > 4 and adjust *p*-value< 0.01.

**Fig. 5:**
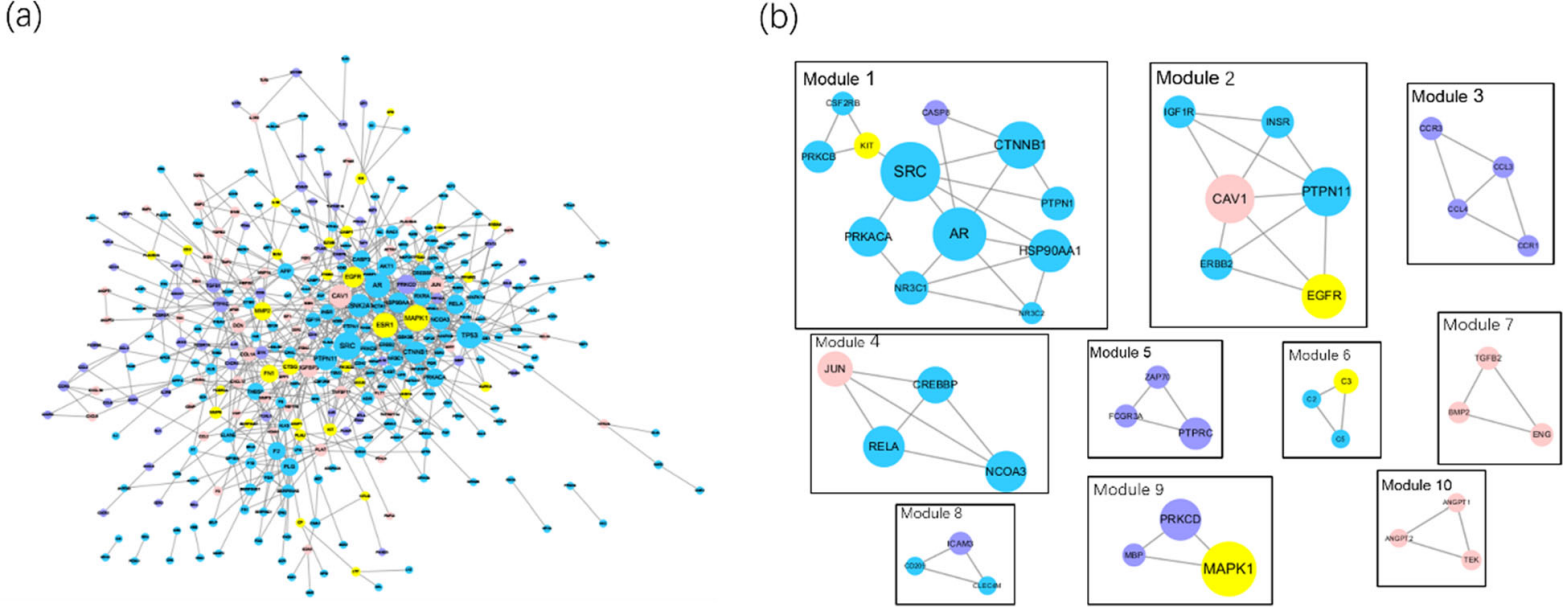
PPIs network of YQCP and DEGs. The blue nodes stand for formula targets; the yellow nodes stand for the formula targets overlapped with DEGs; the purple nodes stand for down-expressed DEGs; the pink nodes stand for up-expressed DEGs. **a**; The whole PPIs network. **b**: sub-cluster with source > 3

Analysis result from microarray obtained 1579 DEGs. Five hundred ninety-one RA-related genes were embodied in Genecards with score > 5. One hundred ninety-eight proteins translated from overlapped genes in these two data were finally chosen as therapy-related target. Association between therapy-related proteins and formula targets were analyzed using PPIs network (Fig. 5a) and MCODE plugin was applied to figure out the stable sub-clusters in the whole PPIs and 10 sub-clusters were meet with the criteria that cluster score > 3 (Fig. 5b). Formula targets either overlapped with disease genes or have the high degree were chosen as the candidate hub targets, such as SRC in cluster 1 and EGFR in cluster 2. Literature mining then verified the hub targets based on whether they contribute to the therapy or pathogenesis of RA. For instance, several recent studies demonstrated that EGFR concentrations are markedly elevated both in serum and synovial fluid in RA patients as contrasted to health controls [22]. Besides, the tissue mRNA expressions of Src kinase were increased, and it’s signaling pathway is active in RA [23]. Targets like abnormal IGF-I production take effect in aberrant osteoclastic activation and angiogenesis, and IGF inhibition is beneficial for the treatment of RA [24]. As the importance of complement and MMPs is disscussed in the function analysis, we finally defined C2, C3, C5, MMP2, MMP9, SRC, KIT, IGF1R and EGFR as hub targets.

### Molecular docking analysis

**Fig. 6:**
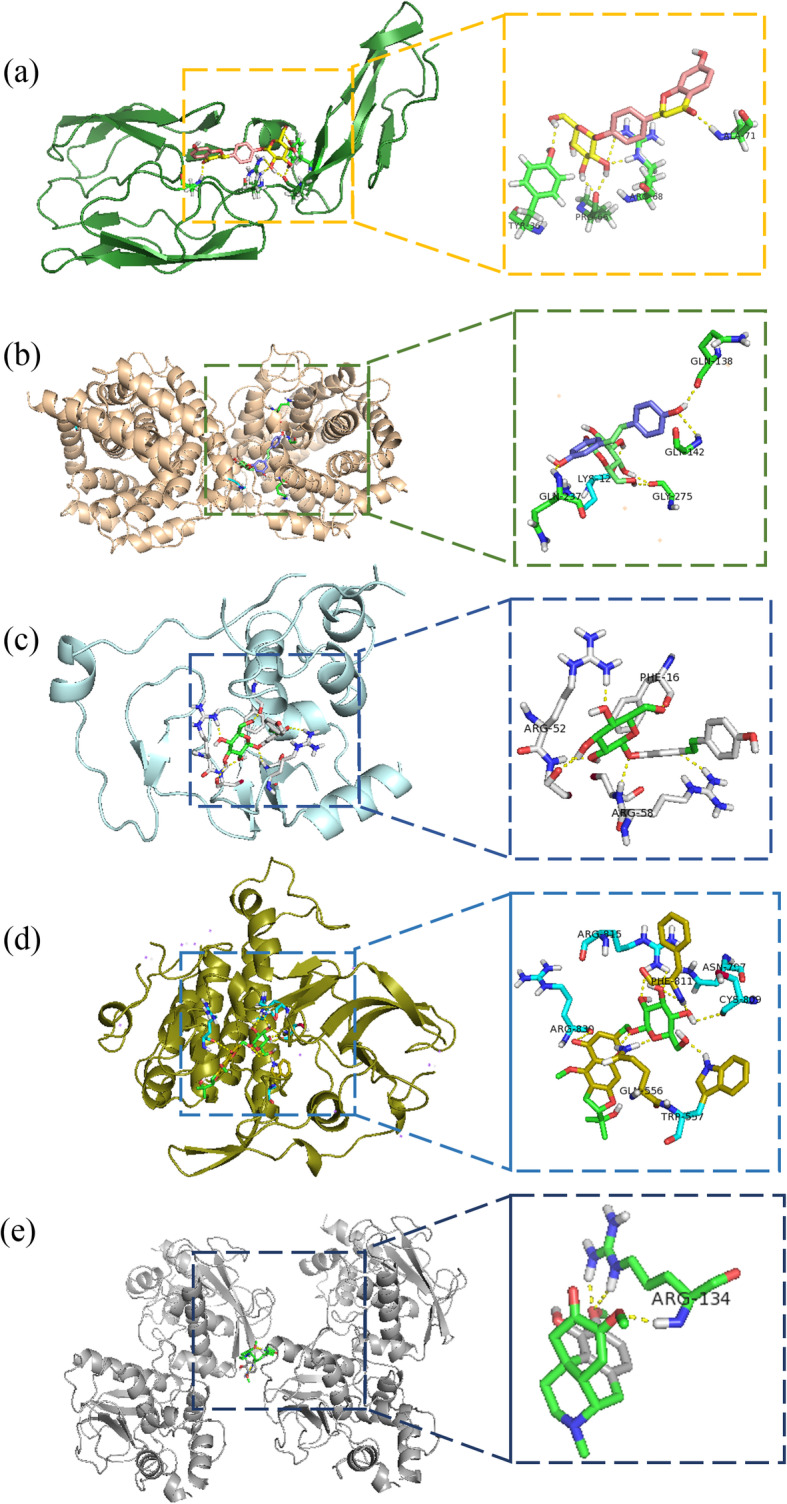
Molecular docking patterns of main ingredients and key targets of YQCP

To ascertain the feasibility of YQCP in targeting the hub proteins, we carried out molecular docking analysis on the five identified ingredients and 9 hub targets. 2D structure of the ingredients and crystal structure of the 9 targets were downloaded. PDB id of C2, C3, C5, MMP2, MMP9, SRC, KIT, IGF1R and EGFR are respectively 3ERB, 3D5R, 3CU7, 1KS0, 5TH6, 3GEQ, 3G0E, 2DSP and 1JL9. Except C5 and MMP2, the rest 7 targets were all showed good affinity with the main ingredients (Table 2). We chose hydroxychloroquine as control. The average affinity between the 5 main component and targets is better than hydroxychloroquine. Therein, combination success of sinomenine is highest among the 5 ingredients while combination success of C3 and KIT is highest among the 7 hub targets. Molecular docking diagrams of C2 - liquiritin, C3 - chlorogenic acid, IGF1R - polydatin KIT - prim-O-glucosylcimifugin and MMP9 - sinomenine were showed in Fig. 6.

**Table 2:** The binding energy between targets and moleculars

Target	Chlorogenic acid (kcal·mol^−1^)	Polydatin (kcal·mol^− 1^)	Prim-O-glucosylcim-ifugin (kcal·mol^− 1^)	Sinomenine (kcal·mol^− 1^)	Liquiritin (kcal·mol^− 1^)	Hydroxychloro-quine (kcal·mol^− 1^)
C2	−3.75	−4.90	− 4.13	−5.28	−6.26	−3.45
C3	−7.30	−9.41	−8.18	− 9.42	−9.49	− 4.28
IGF1R	−5.19	−6.48	−4.38	−6.05	−5.27	−3.13
MMP9	−4.09	−4.96	−5.39	−6.21	− 4.47	/
SRC	−5.05	−5.09	−4.40	−6.76	− 5.50	−3.84
KIT	−6.18	−7.35	− 7.36	− 6.80	−7.87	− 6.33
EGFR	− 5.35	− 4.90	− 5.43	−5.21	−4.38	3.13

## Discussion

RA is an autoimmune disorder characterized by lasting articular inflammation and high risk for disability. DMARDS, NSAIDs are still the mainstays of therapy. Biologicals are not widespread because of the expensive medical fee. Rheumatologist have reached an agreement that early identification and treatment is of great importance in RA disease management. In China, TCM prescription has long been an alternative medicine for arthritis including RA. The superiority of TCM is prevention before disease onset.

Cangzhu Fangfeng Tang is an effective traditional formula which was recorded in “Su Wen Bing Ji Qi Yi Bao Ming Ji”. With the combination of traditiaonl medicine, modern pharmacology and clinical practice, Prof. Wen come up with a modified Cangzhu Fangfeng Tang, namely YQCP. Previously, we have demonstrated that preventive treatment of YQCP can effectively delay the occurrence RA and relieve the inflammatory response [8]. Because of the hallmarks of multi-ingredients and multi-targets, it is difficult to uncover the specific mechanism of TCM formula. Recently, network pharmacology and bioinformatics were widely used in predicting the molecular function, providing guidance on experiment design.

In this research, 699 kinds of components were collected from databases. Some of the components have been fully studied, such as chlorogenic acid, prim-O-glucosylcim-ifugin, polydatin, sinomenine and liquiritin. Chlorogenic acid is a dietary polyphenol. Chlorogenic acid and prim-O-glucosylcimifugin both play important roles in anti-inflammatory [27, 28]. Polydatin and sinomenine promote the osteogenic differentiation and inhibit NLRP3 inflammasome [29, 30]. Moreover, clinical trial confirmed that sinomenine slow down the progression of RA. We believe that the mechanism of prophylactic treatment of RA may be based on the clearance of the early inflammatory environment and adjustment of ratio of osteoblast/osteoclast.

Two databases, i.e. TCMID and Pharmmapper, which used different ways to predict chemical targets was applied to gather the formula targets. Four hundred thirteen overlapped targets in those two databases was adopted as targets of YQCP. GO analysis indicated that YQCP may take effect through three aspects: mature B cell apoptotic process, complement alternative pathway activation and enzymes activity. Serum anticitrullinated protein antibodies (ACPA), which is produced by B cells, can be detected 10 years before final diagnosis of RA [31, 32]. As ACPA precede the typical clinical manifestation, how ACPA generated and accumulated seems to be one of the answers for the pathogenesis of the early onset of RA. Overproduction of mature B cells and B cell-maturation antigen favor towards the production of ACPA contributes to the aggravation of RA [33–35]. Moreover, B cell is an independent factor impacting curative effect [36, 37]. Therefore, intervention on B cells may explain the prophylactic treatment mechanism of YQCP on RA. Complement alternative pathway activation is involved in decay-accelerating activity of B cells [38]. As shown in Fig. 4, cluster 6 contains three targets, C3, C5 and C2. Therein, both C5 and C3 take part in complement alternative pathway and influence aberrant activation of B cell [39, 40]. The GO result shows that YQCP could modulate the activity of some enzymes such as endopeptidase, peroxidase and metallopeptidase. Enzyme inhibitor has been used as therapeutic agent for a long time. Different enzyme plays different roles in the pathogenesis. Among them, endopeptidase is a kind of fibroblast activation protein that takes part in remodeling of tissues at sites of inflammation. The GO item endopeptidase activity involves hub targets of YQCP MMP2, MMP9 and C3. MMP2 and MMP9 is a matrix metallopeptidase. Overexpression of MMP9 facilitate bone erosion [41]. Peroxidase concerns ROS removal. Overproduction of ROS represent the exacerbation of oxidative stress, indicating an on-going inflammation reaction [42].

In addition to GO analysis, PPIs also screen out some hub targets. SRC is a high degree formula target in cluster 1. SRC is a regulator of integrin-mediated adhesion that involved in bone resorption. It is reported that SRC is regulated by ROS in osteoclast differentiation [43]. The inhibitor of KIT, another hub targets in cluster 1, has been utilized for the treatment of RA [44]. EGFR concentrations are markedly elevated both in serum and synovial fluid in RA patients. EGFR transactivation contribute to cartilage destruction and EGFR inhibitor is also an common drug targets for immune disorders [45, 46]. Cluster 3 mainly effect the NF-κB activation. Hence, C2, C3, C5, MMP2, MMP9, SRC, KIT, IGF1R and EGFR were confirmed as potential hub targets in YQCP for RA intervention.

At last, we carried out autodock to figure out the feasibility of YQCP taking effect on hub targets. The result indicated that the main ingredients of YQCP showed high affinity to the hub targets. Moreover, every ingredient can at least success combined with 4 hub targets which substantiated the multi-target effect of the herbs. Among them, sinomenine exhibited the highest affinity.

## Conclusions

**Fig. 7:**
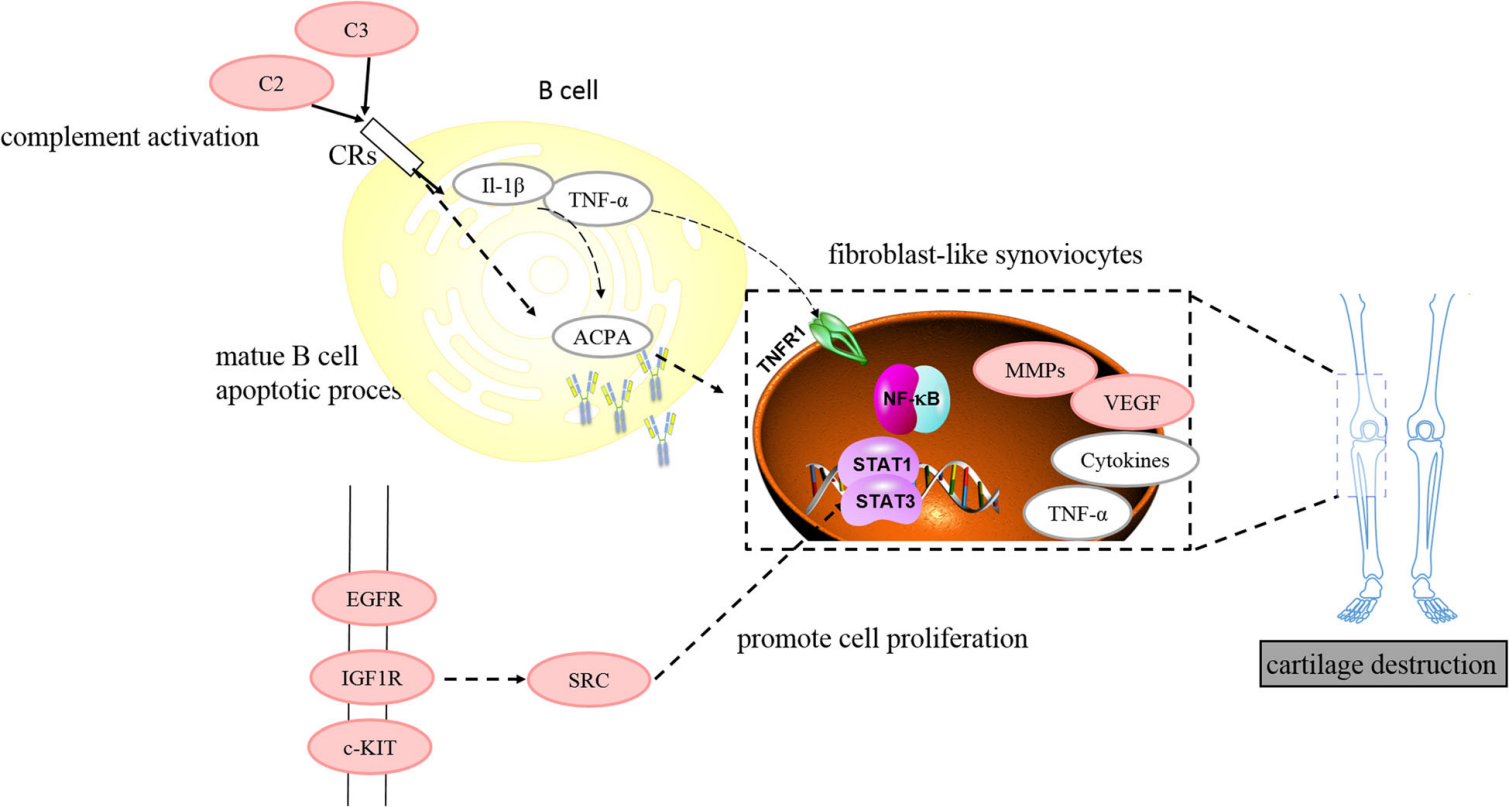
The overall mechanism of the YQCP on RA therapy

n conclusion, multi-targets and multi-ingredients is the hallmark of TCM. As showed in Fig. 7, YQCP, through acting on hub targets such as EGFR, C2,MMP9, may influence the mature of B cells and inhibit B cell-related IgG production, regulate oxidative stress and modulate activity of several enzymes including peroxidase and metallopeptidase to delay the occurrence and progress of RA and benefit the pre-RA or early-RA patients.

## Supplementary Information

**Additional file 1: Figure S1.** LC-MS identify main ingredient. **Table S1.** Identified ingredient through LC-MS. **Table S2.** Batch information of each herbs of herbs. **Table S3.** The ingredient of herbs. **Table S4.** Overlap targets between TCMID and PharmMapper. **Table S5**. Enriched GO items about immune system process of the predicted targets. **Table S6.** Enriched GO items about molecular function of the predicted targets. **Table S7**. Enriched KEGG pathway of the predicted targets.

**Additional file 2.** Targets information of YQCP.

## Data Availability

The datasets generated and/or analysed during the current study are available in the TCMID, http://119.3.41.228:8000/tcmid/; Pharmmapper, http://lilab-cust.cn/pharmmapper/index.html, GEO, https://www.ncbi.nlm.nih.gov/geo, PDB, https://www.rcsb.org/ and Pubchem, https://pubchem.ncbi.nlm.nih.gov/.

## Abbreviations

YQCP: Yunpi-Qufeng-Chushi-Prescription
DEGs: Differentially expressed genes
RA: Rheumatoid arthritis
TLRs: Toll-like receptor
TCM: Traditional Chinese Medicine
GO: Gene Ontology
MMPs: matrix metalloproteinases
PPIs: Protein-protein interactions
