# Supplementary material for: Network analysis indicating the pharmacological mechanism of Yunpi-Qufeng-Chushi-prescription in prophylactic treatment of rheumatoid arthritis
Source: BMC Complement Med Ther. 2021 May 15;21:142. doi: 10.1186/s12906-021-03311-4 (PMC8122573; doi:10.1186/s12906-021-03311-4)
Supplement: Supplementary file 1 — Additional file 1: Figure S1. LC-MS identify main ingredient. Table S1. Identified ingredient through LC-MS. Table S2. Batch information of each herbs of herbs. Table S3. The ingredient of herbs. Table S4. Overlap targets between TCMID and PharmMapper. Table S5. Enriched GO items about immune system process of the predicted targets. Table S6. Enriched GO items about molecular function of the predicted targets. Table S7. Enriched KEGG pathway of the predicted targets. [file 12906_2021_3311_MOESM1_ESM.docx]

Network Analysis Indicating the Pharmacological Mechanism of Yunpi-Qufeng-Chushi-Prescription in prophylactic treatment of Rheumatoid Arthritis

Lin Li^1, #^, Donghai Zhou^2,^ ^#^, Qiuping Liu^1^, Dianming Li^1^, Qiao Wang^1^, Xiaowei Shi^1^, Chengping Wen^1, *^, Lin Huang^1,*^

^1^School of Basic Medical Sciences, Zhejiang Chinese Medical University, 548 Binwen Road, Hangzhou, Zhejiang, 310000, China.

^2^ The Second Affiliated Hospital of Zhejiang Chinese Medical University, Hangzhou, 310005, China.

*****Correspondence and requests for materials should be addressed to C.W. (email: [wengcp@163.com](mailto:wengcp@163.com)) and L.H. (email: [huanglin@zcmu.edu.cn](mailto:huanglin@zcmu.edu.cn)).

^#^ These authors contributed equally to this work.

| **Figure S1 LC-MS identify main ingredient.**  **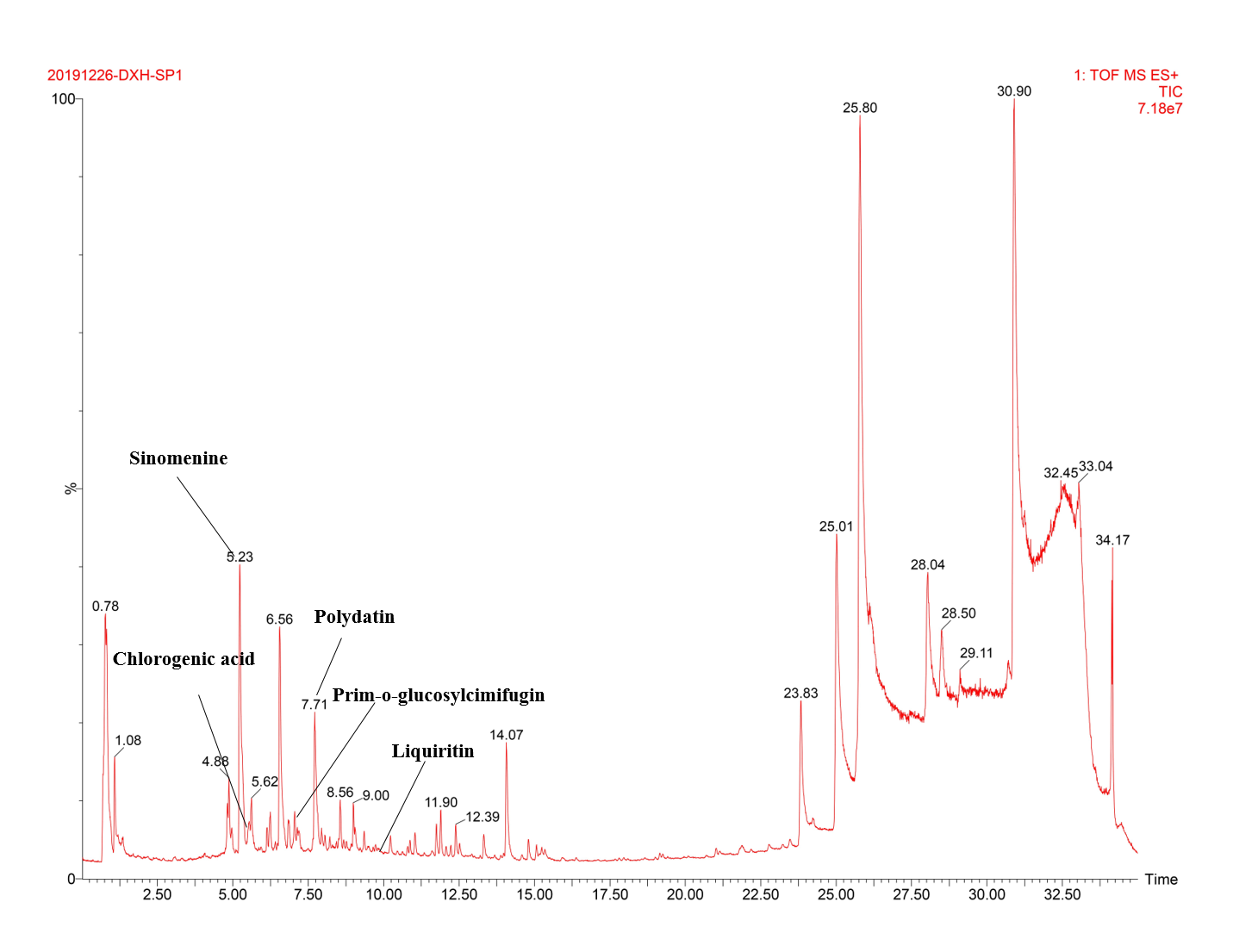**  **Table S1 Identified ingredient through LC-MS.**   \| **Ingredient** \| **Formula** \| **Retention time (min)** \| \| --- \| --- \| --- \| \| Emodin \| C15H10O5 \| 17.52 \| \| D - sesamin \| C20H18O6 \| 16.99 \| \| Licobenzofuran \| C21H22O5 \| 16.71 \| \| Hexadecene \| C16H32 \| 16.39 \| \| farnesyl acetate \| C17H28O2 \| 15.97 \| \| 3 '-O-angelicylhydriferol \| C20H22O6 \| 15.94 \| \| Palmitic acid \| C16H32O2 \| 15.37 \| \| cis-3,7-Dimethyl-2,6-octadienol \| C10H18O \| 15.31 \| \| palustrol \| C15H26O \| 14.83 \| \| 18 β-glycyrrhetinic acid \| C30H46O4 \| 14.81 \| \| Glycyrrhizin \| C42H62O16 \| 14.8 \| \| AtractylenolideⅡ \| C15H20O2 \| 14 \| \| Isoglabrolide \| C30H44O4 \| 13.69 \| \| Formononetin \| C16H12O4 \| 13.52 \| \| Methyl myristate \| C15H30O2 \| 13.5 \| \| Glabrolide \| C30H44O4 \| 12.85 \| \| 6- hydroxyaloe-emodin \| C15H10O6 \| 12.7 \| \| myristic acid \| C14H28O2 \| 12.47 \| \| Bergapten \| C12H8O4 \| 12.24 \| \| anthraglycoside A \| C22H22O10 \| 12.23 \| \| Emodin -8- methyl ether \| C16H12O5 \| 12.23 \| \| Apigenin \| C15H10O5 \| 12.13 \| \| Deoxyglycyrrhetone \| C30H46O3 \| 11.9 \| \| Cholesterol \| C27H46O \| 11.75 \| \| Baicalin methyl ester \| C23H22O11 \| 11.69 \| \| Nodakenitin \| C14H14O4 \| 10.88 \| \| quercetin \| C15H10O7 \| 10.88 \| \| 7,6 '-dihydroxy-3' -methoxy isoflavone \| C16H12O5 \| 10.86 \| \| Hamaudol \| C15H16O5 \| 10.68 \| \| 7-methoxycoumarin \| C10H8O3 \| 10.47 \| \| Licochalcone B \| C16H14O5 \| 10.26 \| \| Trans-N -(4-hydroxyphenylethyl) ferulic acid amide \| C18H19NO4 \| 10.23 \| \| 5-O-methylvisammioside \| C16H18O5 \| 10.19 \| \| Isoliquiritin \| C21H22O9 \| 10.04 \| \| Chrysophanic acid \| C15H10O4 \| 9.95 \| \| liquiritin \| C21H22O9 \| 9.81 \| \| Isoliquiritigenin \| C15H12O4 \| 9.81 \| \| Liquiritigenin \| C15H12O4 \| 9.52 \| \| Isoglycyrrhiza glycoside \| C26H30O13 \| 9.52 \| \| L - menthol \| C10H20O \| 9.31 \| \| Ledebouriellol \| C20H22O7 \| 9.31 \| \| luteolin \| C15H10O6 \| 9.31 \| \| 13,13 a-dideoxy-9, 10-dimethoxy-2, 3-methoxyberberine \| C20H19NO4 \| 9.22 \| \| 3, 5-dicaffeoylquinic acid \| C25H24O12 \| 9.06 \| \| sec-O-Glucosylhamaudol \| C21H26O10 \| 9 \| \| wogonin \| C16H12O5 \| 8.97 \| \| Ω- hydroxyemodin - 8-methyl ether \| C16H12O6 \| 8.96 \| \| naringenin \| C15H12O5 \| 8.95 \| \| Sennoside B \| C21H20O10 \| 8.73 \| \| Licoricone \| C22H22O6 \| 8.6 \| \| Chondrillast-7-enol \| C29H50O \| 8.56 \| \| Dodder glycosides A \| C31H36O16 \| 8.32 \| \| 1，3-O-dicaffeoylquinic acid \| C25H24O12 \| 8.22 \| \| N-(1, 7-dimethoxy-phenyl-2-yl) -acetamide \| C18H17NO3 \| 8.18 \| \| Luteolin - 7-O-α-D-gluconic anhydride \| C21H22O11 \| 8.17 \| \| Kaempferol - 3-O-β-D-glucoside \| C21H20O11 \| 8.07 \| \| kaempferol \| C15H10O6 \| 8.07 \| \| Cimifugin \| C16H18O6 \| 8.06 \| \| Quercetin - 3-O-β-D-glucopyranoside \| C21H20O12 \| 8.05 \| \| Dihydroquercetin \| C15H12O7 \| 8 \| \| Licoflavone A \| C20H18O4 \| 7.94 \| \| (E)-Aldosecologanin \| C34H46O18 \| 7.86 \| \| 3,5,4' -trihydroxy astragalus \| C14H12O3 \| 7.76 \| \| Atractylodin \| C13H10O \| 7.76 \| \| Polydatin \| C20H22O8 \| 7.76 \| \| Licochalcone A \| C21H22O4 \| 7.72 \| \| isoengelitin \| C21H22O10 \| 7.65 \| \| quercetin \| C15H10O7 \| 7.52 \| \| 2,5－Dimethyl－7－hydroxy chromone \| C11H10O3 \| 7.43 \| \| Sesamolin \| C20H18O7 \| 7.38 \| \| 3- feruylquinic acid \| C17H20O9 \| 7.17 \| \| (+)-Syringaresinol \| C22H26O8 \| 7.15 \| \| prim-O-glucosylcimifugin \| C22H28O11 \| 7.02 \| \| Vogeloside \| C17H24O10 \| 6.98 \| \| N-Methylcytisine \| C12H16N2O \| 6.84 \| \| Secoxyloganin \| C17H24O11 \| 6.84 \| \| limonin \| C26H30O8 \| 6.7 \| \| Sweroside \| C17H24O9 \| 6.51 \| \| Epicatechin-3-gallate \| C15H14O6 \| 6.37 \| \| Oxyresveratrol \| C14H12O4 \| 6.36 \| \| Disinomenine \| C38H44N2O8 \| 5.93 \| \| cuscutoside C \| C32H38O17 \| 5.75 \| \| Quercetin-3-o-galactoside-7-o-glucoside \| C27H30O17 \| 5.67 \| \| chlorogenic acid \| C16H18O9 \| 5.58 \| \| Aquilegiolide \| C8H8O3 \| 5.52 \| \| Ferulic acid \| C10H10O4 \| 5.52 \| \| umbelliferone \| C9H6O3 \| 5.31 \| \| 3, 4-dihydroxy cinnamic acid \| C9H8O4 \| 5.31 \| \| Osthol \| C15H16O3 \| 5.24 \| \| Imperatorin \| C16H14O4 \| 5.24 \| \| Acutumine \| C19H24ClNO6 \| 5.21 \| \| Acutumidine \| C18H22ClNO6 \| 4.76 \| \| paeonol \| C9H10O3 \| 4.75 \| \| protocatechnic aldehyde \| C7H6O3 \| 4.48 \| \| tuduranine \| C18H19NO3 \| 4.4 \| \| 3, 5-O-dimethyl-gallic acid \| C9H10O5 \| 3.85 \| \| α-caryophyllene \| C15H24 \| 3.43 \| \| Protocatechuic acid \| C7H6O4 \| 3.21 \| \| deltoin \| C19H20O5 \| 2.45 \| \| Secoxyloganic acid \| C16H22O11 \| 2.44 \| \| 3,4, 5-trimethoxyphenyl-1-O-β-D-glucopyranoside \| C15H22O9 \| 1.89 \| \| 5-hydroxymethylfurfural \| C6H6O3 \| 1.68 \| \| 2-furanic acid \| C5H4O3 \| 1.1 \| \| Secologanic acid \| C16H22O10 \| 1.09 \| \| Malic acid \| C4H6O5 \| 1.06 \| \| citric acid \| C6H8O7 \| 0.87 \| \| 3, 4-dihydroxyphenylethanol-3-O-β-D-glucopyranoside \| C12H16O8 \| 0.82 \| \| 5-hydroxymethylfurfural \| C6H6O3 \| 0.82 \| \| Furfuryl alcohol \| C5H6O2 \| 0.81 \| \| furfuraldehyde \| C5H4O2 \| 0.81 \| \| 2-Methylsuccinic acid \| C5H8O4 \| 0.79 \|   **Table S2.** **Batch information of each herbs of herbs.**   \| **Herbs** \| **Ingredient** \| **Formula** \| **Molecular weight** \| **retention time** \| \| --- \| --- \| --- \| --- \| --- \| \| Lonicera japonica \| chlorogenic acid \| C16H18O9 \| 355.1029 \| 5.31 \| \| Rhizoma Polygoni Cuspidati \| polydatin \| C20H22O8 \| 391.1393 \| 7.75 \| \| Saposhnidoviae Radix \| prim-O-glucosylcimifugin \| C22H28O11 \| 469.171 \| 7.02 \| \| Caulis sinomenii \| sinomenine \| C19H23NO4 \| 330.1705 \| 5.21 \| \| prepared Liquorice root \| liquiritin \| C42H62O16 \| 823.4116 \| 9.76 \|   **Table S3. The ingredient of herbs.** | | | |
| --- | --- | --- | --- | --- | --- | --- | --- | --- | --- | --- | --- | --- | --- | --- | --- | --- | --- | --- | --- | --- | --- | --- | --- | --- | --- | --- | --- | --- | --- | --- | --- | --- | --- | --- | --- | --- | --- | --- | --- | --- | --- | --- | --- | --- | --- | --- | --- | --- | --- | --- | --- | --- | --- | --- | --- | --- | --- | --- | --- | --- | --- | --- | --- | --- | --- | --- | --- | --- | --- | --- | --- | --- | --- | --- | --- | --- | --- | --- | --- | --- | --- | --- | --- | --- | --- | --- | --- | --- | --- | --- | --- | --- | --- | --- | --- | --- | --- | --- | --- | --- | --- | --- | --- | --- | --- | --- | --- | --- | --- | --- | --- | --- | --- | --- | --- | --- | --- | --- | --- | --- | --- | --- | --- | --- | --- | --- | --- | --- | --- | --- | --- | --- | --- | --- | --- | --- | --- | --- | --- | --- | --- | --- | --- | --- | --- | --- | --- | --- | --- | --- | --- | --- | --- | --- | --- | --- | --- | --- | --- | --- | --- | --- | --- | --- | --- | --- | --- | --- | --- | --- | --- | --- | --- | --- | --- | --- | --- | --- | --- | --- | --- | --- | --- | --- | --- | --- | --- | --- | --- | --- | --- | --- | --- | --- | --- | --- | --- | --- | --- | --- | --- | --- | --- | --- | --- | --- | --- | --- | --- | --- | --- | --- | --- | --- | --- | --- | --- | --- | --- | --- | --- | --- | --- | --- | --- | --- | --- | --- | --- | --- | --- | --- | --- | --- | --- | --- | --- | --- | --- | --- | --- | --- | --- | --- | --- | --- | --- | --- | --- | --- | --- | --- | --- | --- | --- | --- | --- | --- | --- | --- | --- | --- | --- | --- | --- | --- | --- | --- | --- | --- | --- | --- | --- | --- | --- | --- | --- | --- | --- | --- | --- | --- | --- | --- | --- | --- | --- | --- | --- | --- | --- | --- | --- | --- | --- | --- | --- | --- | --- | --- | --- | --- | --- | --- | --- | --- | --- | --- | --- | --- | --- | --- | --- | --- | --- | --- | --- | --- | --- | --- | --- | --- | --- | --- | --- | --- | --- | --- | --- | --- | --- | --- | --- | --- | --- | --- | --- | --- | --- | --- | --- | --- | --- | --- | --- | --- | --- | --- | --- | --- | --- | --- | --- | --- | --- | --- | --- | --- | --- | --- | --- | --- | --- | --- | --- | --- | --- | --- | --- |
| **Herb** | **Ingredient** | **Herb** | **Ingredient** |
| cang zhu | 10-epiatractyloside a | qing feng teng | acutuminine |
| cang zhu | atractyloside c | qing feng teng | acutumine |
| cang zhu | eudesmol | qing feng teng | acutumidine |
| cang zhu | scopoletin β-d-xylopyranosyl-(1→6)-β-d-glucopyranoside | qing feng teng | sinoacutine |
| cang zhu | bata-caryophyllene | qing feng teng | sinomenine |
| cang zhu | uridine | qing feng teng | (?)-salutaridine |
| cang zhu | elemol | qing feng teng | magnograndiolide |
| cang zhu | elemicin | qing feng teng | stigmasterol |
| cang zhu | elenolide | qing feng teng | (?)-syringaresinol |
| cang zhu | β-elemene | qing feng teng | dauricumine |
| cang zhu | 2-(8-methyl-2,8-dihydroxy-9-oxo-2-hydroxymethylbicyclo[5.3.0]decan-7-yl)isopropanol glucoside | qing feng teng | magnoflorine |
| cang zhu | atractylodinol | qing feng teng | bianfugenine |
| cang zhu | δ-guaiene | qing feng teng | disinomenine |
| cang zhu | 2-furaldehyde | qing feng teng | dispegatrine |
| cang zhu | 2-(8-methyl-2,8,9-trihydroxy-2-hydroxymethyl-bicyclo[5.3.0]decan-7-yl)isopropanolglucoside | qing feng teng | methylhexadecanate |
| cang zhu | cis-atractyloside i | qing feng teng | tufulingoside |
| cang zhu | atractylodin | qing feng teng | tuduranine |
| cang zhu | (2r,3r,5r,7r,10s)-atractyloside g 2-o-β-d-glucopyranoside | qing feng teng | gamma-sitosterol |
| cang zhu | atractyloside i | qing feng teng | 14-methyl hexadecanoic acid |
| cang zhu | atractylenolide i | qing feng teng | tetrahydropalmatine |
| cang zhu | atractylenolide ii | qing feng teng | sinomendine |
| cang zhu | (1s,4s,5s,7r,10s)-10,11,14-trihydroxyguai-3-one 11-o-β-d-glucopyranoside | qing feng teng | 8,14-dihydros-alutaridine |
| cang zhu | (+)-eudesma-4(15),7(11)-dien-8-one | qing feng teng | stigmasterol-beta-d-glucoside |
| cang zhu | eudesobovatol a | qing feng teng | michelenolide |
| cang zhu | (1s,4s,5r,7r,10r)-11,14-dihydroxyguai-3-one11-o-β-d-glucopyranoside | qing feng teng | (+)-suyringaresinol-di-o-beta-d-glucoside |
| cang zhu | β-eudesmol | qing feng teng | sinomontanine d |
| cang zhu | α-eudesmol | qing feng teng | 6-O-demethyl-menisporphine |
| cang zhu | 12,14-diacetate-2E,8E,10E-trien-4,6-diyn-1-ol | qing feng teng | β-sitosterol |
| cang zhu | icariside f2 | qing feng teng | beta-sitosterol |
| cang zhu | (1s,5r,7r,10r)-secoatractylolactone11-o-β-d-glucopyranoside | qing feng teng | sinalbin |
| cang zhu | (2e)-2-decene-4,6-diyne-1,8-diol 8-o-β-d-apio-furanosyl-(1→6)-β-d-glucopyranoside | qing feng teng | dl-sy-ringaresinol |
| cang zhu | hinesol | qing feng teng | Tip tetradrine |
| cang zhu | alpha-chamigrene | qing feng teng | Bat Gebo phenol base |
| cang zhu | adeninenucleoside | qing feng teng | dauriporphinoline |
| cang zhu | atractulodin | qing feng teng | stepholidine |
| cang zhu | atractylon | qing feng teng | (-)-8-oxotetrahydrothalifendine |
| cang zhu | 3beta-acetoxy-atractylon | qing feng teng | (-)-oxoisocorypalmine |
| cang zhu | gamma-selinene | qing feng teng | Tetrahydro table berberine |
| cang zhu | 2-furancarboxylic acid | qing feng teng | stepharanine |
| cang zhu | beta-humulene | qing feng teng | stepharine |
| cang zhu | 6,6'-dimethoxygossypol | qing feng teng | sinactine |
| cang zhu | β-caryophyllene | qing feng teng | isosinomenine |
| cang zhu | tr-saponin a | qing feng teng | isositsivikine |
| cang zhu | tryptophan | qing feng teng | Dodecanoate |
| cang zhu | 3β-acetoxy-atractylon | qing feng teng | michelalbine |
| cang zhu | syringin | tu fu ling | isoengeletin |
| cang zhu | guaiol | tu fu ling | epicatechin |
| cang zhu | 2-methyl-4-(1,1-dimethylethyl)phenol | tu fu ling | 3-o-caffeoylshikimic acid |
| cang zhu | α-guaiene | tu fu ling | 5-o-caffeoylshikimicacid |
| cang zhu | α-humulene | tu fu ling | 2,4,6-trihydroxyacetophenone-2,4-di-o-β-d-glucopyranoside |
| cang zhu | β-chamigrene | tu fu ling | neoastib-lin |
| cang zhu | (2e,8 e)-2,8-decadiene-4,6-diyne-1,10-diol 1-o-β-d-glucopyranoside | tu fu ling | smitilbin |
| cang zhu | atractyloside a 14-o-β-d-fructofuranoside | tu fu ling | 3,4,5-trimethoxyphenyl-1-O-B-D-glucopyranoside |
| cang zhu | atractyloside g | tu fu ling | smiglabrin,Smi1 |
| cang zhu | atractyloside e | tu fu ling | 3,4-two hydroxy benzene ethanol-3-O-B-D- glucopyranoside |
| cang zhu | atractyloside d | tu fu ling | 3,4,5- trimethoxyphenyl-1-O- B-D- furan apioside base-(1Y6)B-D- glucopyranoside |
| cang zhu | 3β-hydroxyatractylone | tu fu ling | astilbin |
| cang zhu | atractyloside b | tu fu ling | (2R, 3R)- taxifolin-3-'O-B-D- glucopyranoside |
| cang zhu | beta-eudesmol | tu fu ling | isobaimuxinol |
| cang zhu | atractyloside a | tu fu ling | amylum |
| cang zhu | atractylone | tu fu ling | dioscin |
| cang zhu | (5r,7r,10s)-isopterocarpolon β-d-gluco-pyranoside | tu fu ling | (-)-epicatechin |
| cang zhu | 1-3alpha,6beta-ditigloyloxytropane | tu fu ling | daucosterol |
| cang zhu | β-maaliene | tu fu ling | 7,6'-dihydroxy-3'-methoxyisoflavone |
| cang zhu | 2,6-ditertbutyl-4methyl phenol | tu fu ling | tigonin |
| cang zhu | diterbutyl phthalate | tu fu ling | 4,7-dihydroxy-5-methoxyl-6-methyl-8-formyl-flavan |
| cang zhu | methyl 3,4,5-trimethoxycinnamate | tu fu ling | isoengelitin |
| cang zhu | β-selinene | tu fu ling | anthemisol |
| cang zhu | 1beta-hydroxybaccatin i | tu fu ling | tufulingoside |
| cang zhu | (1s,4s,5s,7r,10r)-10,11,14-trihydroxyguai-3-one 11-o-β-d-glucopyranoside | tu fu ling | tulipalin |
| cang zhu | (+)-maalioxide | tu fu ling | syrionylglycerol-beta-syringaresinol |
| fang feng | panaxynol | tu fu ling | trans-resveratrol |
| fang feng | panaxydol | tu fu ling | isoastiblin |
| fang feng | bergapten | tu fu ling | neoisoastiblin |
| fang feng | 4-O-β-D-glucosyl-5-O-methylvisamminol | tu fu ling | hexose |
| fang feng | hamaudol | tu fu ling | succinic acid |
| fang feng | 3′-O-angeloyhamaudol | tu fu ling | ferulaic acid |
| fang feng | 3′-O- acetylhamaudol | tu fu ling | isoeruboside b |
| fang feng | sec-o-glucosylhamaudol | tu fu ling | taxifolin |
| fang feng | ledebouriellol | tu fu ling | enhydrin |
| fang feng | 5-O-methylvisamminol | tu fu ling | smiglaside d |
| fang feng | cimifugin | tu fu ling | smiglaside c |
| fang feng | prim-o-glucosylcimifugin | tu fu ling | smiglaside b |
| fang feng | psoralen | tu fu ling | smiglaside a |
| fang feng | xanthotoxin | tu fu ling | syringicacid |
| fang feng | imperation | tu fu ling | dihydrokaempferol-3-o-α-l-rhamnopyranoside |
| fang feng | isoimperation | tu fu ling | smilagenin |
| fang feng | phelloptern | tu fu ling | smiglaside e |
| fang feng | isobergapten | tu fu ling | isoastilbin b |
| fang feng | deltoin | tu fu ling | Butyl--B-- glucopyranoside |
| fang feng | marmesin | tu fu ling | linoleic acid |
| fang feng | nodakenetin | tu fu ling | Butyl--D-- glucopyranoside |
| fang feng | fraxidin | tu fu ling | Resveratrol-3- O-B-D- glucopyranoside |
| fang feng | isofraxidin | tu fu ling | sodium tauropythocholate |
| fang feng | scopoletin | tu fu ling | resveratrol |
| fang feng | sapodivari | tu fu ling | dihydroquercetin |
| fang feng | anomalin | tu fu ling | dihydroresveratrol |
| fang feng | decurson | tu si zi | riboflavin |
| fang feng | decursinolangelate | tu si zi | neosurugatoxin |
| fang feng | α-pinene | tu si zi | Vitamin A |
| fang feng | hexanal | tu si zi | protein |
| fang feng | pentanol | tu si zi | daucosterol |
| fang feng | hexanol | tu si zi | hyperoside |
| fang feng | octanal | tu si zi | kaenpferol |
| fang feng | nonanal | tu si zi | shu zhi yang gan |
| fang feng | octanol | tu si zi | tu si zi gan |
| fang feng | octanoic Acid | tu si zi | methyl-3-O-β-D-glucopyranosyl-5-hydroxycinnamate |
| fang feng | acetophenone | tu si zi | alkaloid |
| fang feng | 7-octen-4-ol | tu si zi | astragalin |
| fang feng | naphthalene | tu si zi | quercetin-3-l-arabinon-7-d-glucoside |
| fang feng | octadecadienoic acid | tu si zi | campesterol |
| fang feng | falcarinol | tu si zi | shu zhi |
| fang feng | cyclohexene | tu si zi | β-sitosterol |
| fang feng | calacorene | tu si zi | astragalin 6''-O-gallate |
| fang feng | decenal | xu chang qing | paeonol |
| fang feng | decadienal | xu chang qing | lineolone |
| fang feng | 3R-(3α,3aβ,7β,8aα)]-8H-3 | xu chang qing | neocynapanogenin c3-o-β-d-oleandropyranoside |
| fang feng | n-heptane | xu chang qing | neocynapanoside a |
| fang feng | n-octane | xu chang qing | para-hydroxy-acetophenone |
| fang feng | n-caproaldehyde | xu chang qing | sarcovaging a |
| fang feng | 2-heptanone | xu chang qing | sarcostin |
| fang feng | n-nonane | xu chang qing | cis-9,cis-12-linoleic acid |
| fang feng | heptanal | xu chang qing | acetophenone |
| fang feng | α-thujene | xu chang qing | isopaeonol |
| fang feng | camphene | xu chang qing | 7-demethoxytylophorine |
| fang feng | 2-octanone | xu chang qing | 2,4-dihydroxy acetophenone |
| fang feng | benzaldehyde | xu chang qing | 3-methoxyphenol |
| fang feng | β-pinene | xu chang qing | tomentolide a |
| fang feng | myrcene | xu chang qing | tomentogenin |
| fang feng | caprylic aldehyde | xu chang qing | 4-methyl-2-methoxyphenol |
| fang feng | cerotic acid | xu chang qing | anthracene |
| fang feng | vanillic acid | xu chang qing | octanoic acid |
| fang feng | falcarindiol | xu chang qing | ethyl benzene |
| fang feng | glycerol monolinoleate | xu chang qing | hexanoic acid |
| fang feng | glycerol monooleate | xu chang qing | caprylic acid |
| fang feng | β-sitosterol | xu chang qing | nonanoic acid |
| fang feng | daucosterol | xu chang qing | 4-hydroxy-3,5-dimethoxyacetophenone |
| fang feng | D-mannitol | xu chang qing | tetradecanoic acid |
| fang feng | fangfengalpyrimidine | xu chang qing | hexadecanoic acid |
| fang feng | adenosine | xu chang qing | 2-hydroxy-5-methoxyacetophenone |
| fang feng | undulatoside | xu chang qing | 2-methyl naphthalene |
| fang feng | undulatoside A | yi yi ren | rhamnose |
| fang feng | wogonin | yi yi ren | coixenolide |
| fang feng | divaricatol | yi yi ren | coixol |
| fang feng | 5-hydroxy-8-methoxypsoralen | yi yi ren | Arabinose |
| fang feng | clemiscosin A | yi yi ren | lead |
| fang feng | tectochrysin | yi yi ren | glucose |
| hu zhang | falacinol | yi yi ren | triolein |
| hu zhang | chrysophanol | yi yi ren | colchamine |
| hu zhang | luteolin-7-beta-d-glucuronide | yi yi ren | stigmasterol |
| hu zhang | chrysophanol-8-o-beta-d-(6'-o-galloyl)-glucopyranoside | yi yi ren | mannose |
| hu zhang | 1-(3-o-β-d-glucopyranosyl-4,5-dihydroxy-phenyl)-ethanone | yi yi ren | campesterol |
| hu zhang | citreorosein | yi yi ren | α-sitosterol |
| hu zhang | 7-hydroxy-4-methoxy-5-methylcoumarin | yi yi ren | lysine |
| hu zhang | picralinal | yi yi ren | friedelin |
| hu zhang | quercetin-3-rhamnoside-7-glucoside | yi yi ren | glucan7 |
| hu zhang | (+)-catechin | yi yi ren | glucan6 |
| hu zhang | guaijaverin | yi yi ren | glucan5 |
| hu zhang | 1-(3',5'-dihydroxyphenyl)-2-(4''-hydroxy-phenyl)-ethane-1,2-diol | yi yi ren | glucan4 |
| hu zhang | fallacinol | yi yi ren | Trilinolein standard |
| hu zhang | 3,4-dihydroxybenzoicacid | yi yi ren | indene |
| hu zhang | quercetin-5,3-di-d-galactoside | yi yi ren | magnesium |
| hu zhang | cinaroside | yi yi ren | octadecenoic acid |
| hu zhang | 2,6-dihydroxybenzoicacid | yi yi ren | glyceride |
| hu zhang | protocatechuic acid-3-glucoside | yi yi ren | stearic acid |
| hu zhang | fangchinoline | yi yi ren | α-monolinolein |
| hu zhang | gallicacid | yi yi ren | palmitic acid |
| hu zhang | rheochrysidin | yi yi ren | 1,2linoleic acid-3oleic acid- triglyceride |
| hu zhang | 2,3,5,4'-tetrahydroxystilbene-2-o-β-d-glucoside | yi yi ren | valine |
| hu zhang | 3,4,3',5'-tetrahydroxystilbene-3-glucoside | yi yi ren | oleic acid |
| hu zhang | protocatechnoc acid | yi yi ren | manganese |
| hu zhang | 1,3-dihydroxy-2-methoxy xanthone | yi yi ren | arginine |
| hu zhang | (-)-3-hydroxy-4-methoxy-8-9-methylenedioxy pterocarpan | yi yi ren | Galactose |
| hu zhang | (?)-lyoniresinol-2a-sulfate | yi yi ren | calcium |
| hu zhang | citric acid | yi yi ren | isoarborinol |
| hu zhang | questinol | yi yi ren | β-sitosterol |
| hu zhang | questin | yi yi ren | cadmium |
| hu zhang | 4-hydroxy-3-methoxyphenolβ-d-glucopyranoside | yi yi ren | copper |
| hu zhang | (?)-isolariciresinol-2a-sulfate | yi yi ren | glucan2 |
| hu zhang | trans-resveratrol | yi yi ren | glucan3 |
| hu zhang | resveratrol- 3-O-β-D-glucoside | yi yi ren | coixan A |
| hu zhang | anthraglycoside a | yi yi ren | coixan B |
| hu zhang | picied | yi yi ren | coixan C |
| hu zhang | citreorosien | yi yi ren | glucan1 |
| hu zhang | quercetin | yi yi ren | Coixendide |
| hu zhang | physovenine | yi yi ren | feruloylstigmasterol |
| hu zhang | physcion-8-o-β-d-glucopyranoside | yi yi ren | feruloylcampesterol |
| hu zhang | physicion | yi yi ren | octadecadienoic acid |
| hu zhang | physcion | yi yi ren | mercury |
| hu zhang | tryptophan | yi yi ren | leucine |
| hu zhang | torachrysone-8-o-β-d-glucoside | yi yi ren | myristic acid |
| hu zhang | quercetin-3-l-arabinon-7-d-glucoside | yi yi ren | zinc |
| hu zhang | polygalacic acid | yi yi ren | phosphorus |
| hu zhang | 4-methoxy-acetophenone | zhi gan cao | methyl-n-butyl-uralsaponin a esters |
| hu zhang | (+)-catechin-5-o-glucoside | zhi gan cao | glycyrrhisoflavone |
| hu zhang | quillaic acid | zhi gan cao | licoleafol |
| hu zhang | 2-methoxy-6-acetyl-7-methyljuglone | zhi gan cao | licopyranocoumarin |
| hu zhang | flavenol glycoside | zhi gan cao | licoricesaponin a3 |
| hu zhang | 3,4-dihydroxy-5-methoxybenzoicacid methyl ester-4-sulfate | zhi gan cao | licofuranocoumarin |
| hu zhang | emodin-6-glucoside | zhi gan cao | 3-hydroxyglabrol(ii) |
| hu zhang | piceid | zhi gan cao | ononin |
| hu zhang | physciondiglucoside | zhi gan cao | 8-methoxy-5-o-glucoside flavone |
| hu zhang | torachrysone-8-o-beta-d-(6'-oxayl)-glucoside | zhi gan cao | 3'-methoxyglabridin |
| hu zhang | emodin | zhi gan cao | licoricesaponin b2 |
| hu zhang | emodin anthrone | zhi gan cao | licoricesaponin c2 |
| hu zhang | emodin-1-o-β-d-glucopyranoside | zhi gan cao | nicotiflorin |
| hu zhang | reynoutrin | zhi gan cao | gmelofuran |
| hu zhang | 2,5-dimethyl-7-hydroxy chromone | zhi gan cao | ferulic acid |
| hu zhang | anthraquinone | zhi gan cao | glyzaglabrin |
| hu zhang | rhein diglucoside | zhi gan cao | hispidulin |
| hu zhang | rhein | zhi gan cao | 3-o-acetyl-glycyrrhetinicacid |
| hu zhang | apigenin | zhi gan cao | glabrolide |
| hu zhang | anthraglycoside b | zhi gan cao | glycyrrhetinic acid |
| hu zhang | polygonin | zhi gan cao | 2,4,4'-trihydroxychalcone |
| hu zhang | resveratrol | zhi gan cao | gancaonin p-3'-methylether |
| hu zhang | polydatin | zhi gan cao | gancaonin x |
| hu zhang | quercetin-3-β-d-xylopyranoside | zhi gan cao | sigmoidin b |
| hu zhang | 3,5-dimethyl-4-methoxybenzoic acid | zhi gan cao | glycyrrhetinicacid |
| hu zhang | quercitrin | zhi gan cao | neoliquiritin |
| jin yin hua | 1-hexanol | zhi gan cao | 3,4-dicaffeoyl-5-(3-hydroxy-3-methyl)glutaroyl quinic acid |
| jin yin hua | loniceracetalides b | zhi gan cao | isoramanone |
| jin yin hua | isochlorogenic acid | zhi gan cao | n-tricosane |
| jin yin hua | new triterpennoid glycoside | zhi gan cao | isoononin |
| jin yin hua | benzyl cyanide | zhi gan cao | 8-methyl-10-hydroxylycoctonine |
| jin yin hua | 2-furaldehyde | zhi gan cao | (e)-1-[2,4-dihydroxy-3-(3-methyl-2-butenyl)phenyl]-3-(2,2-dimethyl-8-hydroxy-2h-benzo-pyran-6-yl)-2-propen-1-one |
| jin yin hua | ioniceroside c | zhi gan cao | methyl-24-hydroxyglycyrrhetate |
| jin yin hua | chlorogenin | zhi gan cao | (e)-1-[2,4-dihydroxy-3-(3-methyl-2-butenyl)phenyl]-3-(4-hydroxy-3-[3-methyl-2-butenyl)phenyl]-2-propen-1-one |
| jin yin hua | chlorogenicacid | zhi gan cao | methylglycyrrhetate |
| jin yin hua | farnesal | zhi gan cao | methylglyoxal |
| jin yin hua | gamma-sitosterol | zhi gan cao | licorisoflavan a |
| jin yin hua | benzyl alcohol | zhi gan cao | licoricone |
| jin yin hua | alpha-terpineol | zhi gan cao | isoliquiriligenin |
| jin yin hua | loganoside | zhi gan cao | kanzonol l |
| jin yin hua | citronellol | zhi gan cao | kanzonol k |
| jin yin hua | citronellyl acetate | zhi gan cao | 18alpha-glycyrrhetinic acid |
| jin yin hua | eugenol methyl ether | zhi gan cao | licoricesaponin e2 |
| jin yin hua | l-phenylalaninosecologanin | zhi gan cao | licoricesaponin d3 |
| jin yin hua | eugenol | zhi gan cao | licoricesaponin g2 |
| jin yin hua | 7-o-(4-β-d-glucopyranosyloxy-3-methoxy-benzoyl)secologanolicacid | zhi gan cao | licoricesaponin f3 |
| jin yin hua | α-pinene | zhi gan cao | licoricesaponin j2 |
| jin yin hua | 6'-o-(7α-hydroxyswerosyloxy)loganin | zhi gan cao | licoricesaponinh2 |
| jin yin hua | β-sitosterol-β-d-glucoside | zhi gan cao | licoricidin |
| jin yin hua | delta-terpineol | zhi gan cao | licoricesaponin k2 |
| jin yin hua | loniflavone | zhi gan cao | licoisoflavone |
| jin yin hua | lonicerin | zhi gan cao | liquiritin |
| jin yin hua | loniceracetalide b | zhi gan cao | liquiritigenin-7,4'-diglucoside |
| jin yin hua | macrocarpal a | zhi gan cao | liquiritigenin4'-o-β-d-apio-d-furanosyl(1→2)-β-d-glucopyranoside |
| jin yin hua | shuang-hua-chun | zhi gan cao | liquiritigenin |
| jin yin hua | trans-2-hwxwnoic acid | zhi gan cao | gancaonin f |
| jin yin hua | farnesol | zhi gan cao | gancaonin i |
| jin yin hua | hederagenin 3-o-arabinoside | zhi gan cao | gancaonin b |
| jin yin hua | farnesyl acetate | zhi gan cao | gancaonin c |
| jin yin hua | inositol-b | zhi gan cao | gancaonin d |
| jin yin hua | nerol | zhi gan cao | gancaonin e |
| jin yin hua | (3r)-4'-methoxy-2',3,7-trihydroxyisoflavanone | zhi gan cao | tetrahydropalmatine |
| jin yin hua | hederagenin | zhi gan cao | rutin |
| jin yin hua | benzyl benzoate | zhi gan cao | ruvoside |
| jin yin hua | benzyl ethyl alcohol | zhi gan cao | umbelliferone |
| jin yin hua | linamarin | zhi gan cao | lupiwighteone |
| jin yin hua | 1,1'-bicyclohexyl | zhi gan cao | narwedine |
| jin yin hua | inositol c | zhi gan cao | glycyrrhizin |
| jin yin hua | inositol b | zhi gan cao | isogosferol |
| jin yin hua | inositol | zhi gan cao | isoglycyrol |
| jin yin hua | linalyl oxide | zhi gan cao | liquiriligenin |
| jin yin hua | insularine | zhi gan cao | 3'-(γ,γ-dimethylallyl)-kievitone |
| jin yin hua | neochlorogenic acid | zhi gan cao | formononetin-7-glucoside |
| jin yin hua | dibutyl phthalate | zhi gan cao | glycyrol |
| jin yin hua | ethyl palmitate | zhi gan cao | schaftoside |
| jin yin hua | luteolin | zhi gan cao | uralstilbene |
| jin yin hua | chlorogenic acid | zhi gan cao | phaseollinisoflavan |
| jin yin hua | linalool | zhi gan cao | phebalosin |
| jin yin hua | menthyl acetate | zhi gan cao | uralenol |
| jin yin hua | β-sitosterol | zhi gan cao | uralenneoside |
| jin yin hua | inositol-c | zhi gan cao | uralsaponin a |
| jin yin hua | caffeicacid | zhi gan cao | uralenol-3-methylether |
| jin yin hua | beta-pinene | zhi gan cao | uralenin |
| jin yin hua | 2-heptanol | zhi gan cao | uralene |
| jin yin hua | chrysoeriol | zhi gan cao | 5,6,7,8-tetrahydro-4-methylquinoline |
| jin yin hua | stigmasterol-beta-d-glucoside | zhi gan cao | neoisopulegol |
| jin yin hua | isochlorogenic acid b | zhi gan cao | neoisoliquiritin |
| jin yin hua | luteolin 7-o-beta-d-galatoside | zhi gan cao | astragalin |
| jin yin hua | loniceracetalide a | zhi gan cao | 5,6,7,8-tetrahydro-2, 4-dimethylquinoline |
| jin yin hua | caffeic acid | zhi gan cao | monoammonium glycyrrhizinate |
| jin yin hua | macranthoidin a | zhi gan cao | β-sitosterol |
| jin yin hua | macranthoidin b | zhi gan cao | tetrahydroharmine |
| jin yin hua | macranthoin f | zhi gan cao | dibutyl uralsaponin a ester |
| jin yin hua | macranthoside a | zhi gan cao | isoschaftoside |
| jin yin hua | macranthoside b | zhi gan cao | 4'-o-methylglabridin |
| jin yin hua | 3'-o-methyl loniflavone | zhi gan cao | glycyrrhizic acid |
| jin yin hua | pentanoic acid | zhi gan cao | licoflavone |
| jin yin hua | methyllinolenate | zhi gan cao | ethyl-n-buthy-uralsaponin a esters |
| jin yin hua | methyl linoleate | zhi gan cao | hispaglabridin b |
| jin yin hua | stigmasterol | zhi gan cao | hispaglabridin a |
| jin yin hua | 2'-o-[β-d-apiofuranosyl(1→2)-β-d-glucopyra-nosyl]isoliquiritigenin | zhi gan cao | 6,8-bis(c-β-glucosyl)-apigenin |
| jin yin hua | i-linalool | zhi gan cao | 3-hydroxyglabrol |
| jin yin hua | 3-methyl-2-(2-pentenyl)-2-cyclopenten-1-one | zhi gan cao | urea |
| jin yin hua | phenethyl alcohol | zhi gan cao | corylifolinin |
| jin yin hua | loganicacid | zhi gan cao | ganoderic acid a |
| jin yin hua | isochlorogenicacid | zhi gan cao | isotrifoliol |
| jin yin hua | isochlorogenicacid a | zhi gan cao | isotrilobine |
| jin yin hua | 3-methyl butanone | zhi gan cao | berniarin |
| jin yin hua | 2-methyl-1-butanol | zhi gan cao | isoliquiritin |
| jin yin hua | nerolidol | zhi gan cao | isolobelanine |
| jin yin hua | loganin | zhi gan cao | isoliquiritigenin |
| jin yin hua | carvacrol acetate | zhi gan cao | uralsaponin b |
| jin yin hua | carvacrol | zhi gan cao | gamma-sitosterol |
| jin yin hua | (e)-aldosecologanin | zhi gan cao | ononitol |
| jin yin hua | (z)-aldosecologanin | zhi gan cao | sinapic acid |
| jin yin hua | benzaldehyde | zhi gan cao | isoorientin |
| jin yin hua | methyl palmitate | zhi gan cao | neohancoside a |
| jin yin hua | 2-heptadecanone | zhi gan cao | neouralenol |
| jin yin hua | 3-o-beta-d-glucopyranosyl-(1-3)-alpha-l-rhamnopyranosyl-(1-2)-alpha-l-arabinopyranosyl hederagenin 28-o-beta-d-gluco-pyranosyl-(1-6)-beta-d-glucopyranosyl ester(vii) | zhi gan cao | neowilforine |
| jin yin hua | hexanoic acid | zhi gan cao | 3,3'-dimethylquercetin |
| jin yin hua | geraniol | zhi gan cao | dimethyl sebacate |
| jin yin hua | geranyl acetate | zhi gan cao | narcissin |
| jin yin hua | phenylacetaldehyde | zhi gan cao | methyl linoleate |
| jin yin hua | ethyl linolenate | zhi gan cao | glycyroside |
| jin yin hua | 2-methylbutanoic acid | zhi gan cao | isoquercitrin |
| jin yin hua | isoeugenol | zhi gan cao | 18beta-glycyrrhetinic acid |
| jin yin hua | 1-hexene | zhi gan cao | methyl 3-o-beta-d-glucopyranosyl polygalacate |
| jin yin hua | beta-sitosterol-3-o-beta-d-xylopyranoside | zhi gan cao | gloeosteretriol |
| jin yin hua | ethyl-p-digallate | zhi gan cao | glycyrin |
| jin yin hua | ethylpalmitate | zhi gan cao | glycyphyllin |
| jin yin hua | methyl pentose(i) | zhi gan cao | glycycoumarin |
| jin yin hua | α-terpineol | zhi gan cao | lensinine |
| kun ming shan hai tang | triptonolide | zhi gan cao | vicianin |
| kun ming shan hai tang | (+)-catechin | zhi gan cao | alpha-trihydroxy coprostanic acid |
| kun ming shan hai tang | wilsonine | zhi gan cao | isolicoflavonol |
| kun ming shan hai tang | wilforgine | zhi gan cao | methyl 18α-hydroxyglycyrrhetate |
| kun ming shan hai tang | triptonoditerpenic acid | zhi gan cao | isoliensinine |
| kun ming shan hai tang | l-epigallocatechin | zhi gan cao | methyl-24-hydroxy-11-deoxoglycyrrhetate |
| kun ming shan hai tang | procyanidin c | zhi gan cao | methyl 2-hydroxy-3,4-dimethoxy benzoate |
| kun ming shan hai tang | hyponine d | zhi gan cao | licobenzofuran |
| kun ming shan hai tang | abrusgenicacid | zhi gan cao | licobichalcone |
| kun ming shan hai tang | triptolidenol | zhi gan cao | licoisoflavaone |
| kun ming shan hai tang | triptonide | zhi gan cao | licoricesaponine a3 |
| kun ming shan hai tang | triptolide | zhi gan cao | licoricesaponine c2 |
| kun ming shan hai tang | procyanidin b-4 | zhi gan cao | licoricesaponine d3 |
| kun ming shan hai tang | hypoepistephanine | zhi gan cao | licoricesaponine f3 |
| kun ming shan hai tang | hypodiolide a | zhi gan cao | licoricesaponine g2 |
| kun ming shan hai tang | procyanidin b-23,3'-di-o-gallate | zhi gan cao | licoricesaponine h2 |
| kun ming shan hai tang | hypoglaunine | zhi gan cao | licoricesaponine j2 |
| kun ming shan hai tang | gallocatechin-(4α→8)epicatechin | zhi gan cao | licoricesaponine k2 |
| kun ming shan hai tang | 3-oxo-olean-12-en-29-oicacid | zhi gan cao | glycyrrhisoflavanone |
| kun ming shan hai tang | (+)-gallocatechin-hexacetate | zhi gan cao | gancaonin a |
| kun ming shan hai tang | regelin | zhi gan cao | glycyrrhiza-flavonol a |
| kun ming shan hai tang | triptinin a | zhi gan cao | glycyrrhizicacid |
| kun ming shan hai tang | tripdiolide | zhi gan cao | glyeurysaponin |
| kun ming shan hai tang | tripdiotolnide | zhi gan cao | 2,5-dihydroxymethyl-3,4-dihydroxypyrrolidine |
| kun ming shan hai tang | hypolide | zhi gan cao | liquoric acid |
| kun ming shan hai tang | epigallocatechin-(4bata-8)-epicatechin-3-o-gallate ester | zhi gan cao | liquiritigenin-7-o-beta-d-(3-o-acetyl)-apiofuranosyl-4'-o-beta-d-glucopyranoside |
| kun ming shan hai tang | 3β-acetoxyolean-12-en-28-oicacid | zhi gan cao | glisoflavanone |
| kun ming shan hai tang | procyanidin b4 | zhi gan cao | glycyrrhetol |
| kun ming shan hai tang | wilfotrine | zhi gan cao | glyarallin b |
| kun ming shan hai tang | triptonoterpenol | zhi gan cao | neomatatabiol |
| kun ming shan hai tang | (+)-catechin-5-o-glucoside | zhi gan cao | 2-methyl-1,3,6-trihydroxyanthraquinone |
| kun ming shan hai tang | triptotriterpenicacid a' | zhi gan cao | 3-methyl-6,7,8-trihydropyrrolo[1,2-a]pyrimidin-2-one |
| kun ming shan hai tang | triptofordin a | zhi gan cao | licochalcone a |
| kun ming shan hai tang | abrusgenic acid | zhi gan cao | licocoumarone |
| kun ming shan hai tang | triptotin | zhi gan cao | formononetin |
| kun ming shan hai tang | hyponine f | zhi gan cao | 3-o-[β-d-glucuronopyranosyl-(1→2)-o-β-d-glucuronopyranosyl]-24-hydroxyglabrolide |
| kun ming shan hai tang | hyponine e | zhi gan cao | glyurallin a |
| kun ming shan hai tang | tripterine | zhi gan cao | glyyunnanprosapogenin d |
| kun ming shan hai tang | Triptonoditerpenic acid | zhi gan cao | glyuranolide |

**Table S4. Overlap targets between TCMID and PharmMapper.**

| **Target** | **Target** | **Target** | **Target** |
| --- | --- | --- | --- |
| ACHE | GSR | GLO1 | CGA |
| ACPP | GSTP1 | GM2A | CHI3L1 |
| ACR | GSTT2B | MMP2 | CTNNB1 |
| ACTA1 | HAGH | NR1I3 | DNMT1 |
| ACVR2B | HEXB | PSAP | GPX3 |
| ADK | HINT1 | QPCT | NUDT5 |
| ALB | HMGCR | ALOX15 | AKR7A2 |
| CCND1 | HMOX1 | AOC3 | HBB |
| CHAT | HSD11B1 | C3 | CNP |
| CPSF3 | HSD17B1 | CPB2 | F5 |
| DHRS4 | HSP90AA1 | FOLH1 | CASQ2 |
| ERCC1 | IL2 | GALNT1 | EFNA1 |
| ESR2 | IMPA1 | HLA-B | PYGM |
| FABP2 | IMPDH1 | KLK3 | S100A12 |
| FGA | IMPDH2 | MDH2 | PPY |
| GALK1 | INSR | NGLY1 | MDH1 |
| GALM | KDR | PNLIP | ADSL |
| IGF1R | KIT | THBS1 | CYP7A1 |
| IYD | LCN2 | TRIM21 | DCC |
| MB | LDHB | ACE2 | IDH1 |
| ME1 | LGALS2 | ADORA2A | NME3 |
| NLGN1 | LGALS3 | FGG | NME4 |
| NOTCH1 | LTA4H | MAOA | RAC3 |
| NR3C2 | MAN1B1 | RFK | AANAT |
| PARP1 | MAOB | CREBBP | ANXA1 |
| RHO | MAP2K1 | ERBB2 | CD2 |
| SULT2A1 | MAPK1 | GYG1 | CSF2RB |
| THTPA | MAPK14 | LDHC | ODC1 |
| TLR1 | MAPK8 | MASP2 | PTGDS |
| ALOX12 | MIF | PDX1 | SERPINA5 |
| AR | MME | APEX1 | BCL2A1 |
| AZGP1 | MMP9 | CA14 | CYP2E1 |
| C8A | NOS2 | CPO | SULT1B1 |
| CUL5 | NOS3 | HBA1 | FZD8 |
| CYP2A13 | NQO1 | HK2 | HYAL1 |
| FABP1 | NR1I2 | HLA-DRB1 | ARF6 |
| FCGRT | NR3C1 | HMGCS1 | DPP6 |
| LIFR | NT5M | IDH2 | IL10 |
| LYZ | PCK1 | IL5 | NFATC1 |
| NCOA3 | PDE5A | LDHA | PIP |
| PMM1 | PGF | PA2G4 | IL1B |
| PMP2 | PGR | PTGS2 | INS |
| RBP4 | PIK3CG | RAC1 | KIRREL3 |
| SH3BGRL | PIM1 | RBP1 | PTPRM |
| TP53 | PKLR | RBP2 | C2 |
| UEVLD | PLA2G10 | CLEC4M | GRIN2A |
| ACADM | PLA2G2A | B2M | NEO1 |
| ADAM17 | PLAU | CD1B | APP |
| ADAM33 | PNP | EGF | AKR7A3 |
| ADH5 | PPARA | HEXA | MAN1A1 |
| AHCY | PPARG | LTF | PCK2 |
| AKR1B1 | PPP5C | NAGK | VEGFA |
| AKR1C1 | PRKACA | S100B | ALDH1A2 |
| AKR1C2 | PTPN1 | YAP1 | SIRT3 |
| AKR1C3 | PTPN11 | ADA | RBP5 |
| AKT1 | PYGL | DDC | GALK2 |
| ALDH2 | RARA | GGPS1 | HLA-A |
| AMD1 | RARB | MAN2B1 | CANX |
| AMY1A | REG1A | PTGR2 | FN1 |
| AMY2A | REN | GSTZ1 | GLUL |
| ANG | RHOA | SULT1A1 | ITGAV |
| APRT | RNASE3 | TRDMT1 | RELA |
| AURKA | RXRA | AGT | THBS2 |
| B3GAT1 | S100A9 | AKR1C4 | EXTL2 |
| BACE1 | SELP | C5 | NCF2 |
| BCAT2 | SERPINA1 | CEL | ALDH3A1 |
| BCHE | SOD2 | G6PD | PDF |
| BHMT | SRC | MAPK9 | CDH2 |
| CA2 | SULT1E1 | PTGS1 | EIF4G1 |
| CASP1 | TGM2 | NME2 | CPA4 |
| CASP3 | TPI1 | SELE | ADH6 |
| CD209 | TTR | UMPS | CP |
| CDK2 | UCK2 | LPA | KITLG |
| CES1 | VDR | PLG | NOS1 |
| CFB | YARS | DDX39B | PLA2G1B |
| CFD | ADH1B | GRIK2 | RTN4IP1 |
| CHIT1 | APCS | GRIN3A | PNPT1 |
| CMA1 | APOA2 | GZMB | BCAT1 |
| CSNK2A1 | ARF1 | PCSK1 | GCNT1 |
| CTSL | BLVRB | BCKDHA | TRPM7 |
| CTSS | BMP7 | ECE1 | IMPA2 |
| CYP2C9 | CASP7 | IL6ST | CFTR |
| DCK | CAT | SERPINC1 | GRIN1 |
| DHFR | CCNT1 | CLCNKA | IGF2R |
| DHODH | CPB1 | FCGR2A | STEAP3 |
| DPP4 | CTSD | GMPS | SEMA4D |
| EGFR | DAPK1 | HEBP1 | AGA |
| ELANE | FABP5 | PTH | TXNRD1 |
| EPHA2 | FCAR | SIN3A | ITGB2 |
| ESR1 | GC | TOP2A | MIOX |
| F10 | LGALS7 | MPO | CS |
| F2 | MMP1 | S100A8 | ENO1 |
| F7 | NPR3 | SERPING1 | PRKCB |
| FABP3 | NQO2 | DPYD | PTPRD |
| FABP4 | NUDT9 | ACO1 | CPOX |
| FDPS | PLK1 | ACOT12 | CHIA |
| FKBP1B | RTN4R | CPA1 | DDAH1 |
| GALE | ACE | CRKL | RBKS |
| GBA | C8G | GRIK1 | EIF4EBP1 |
| GCK | CTSG | GRIN3B | RNASE1 |
| GLTP | CYP2C8 | HLA-G | GRM7 |
| GNPDA1 | F11 | PNLIPRP1 |  |
| GP1BA | FABP7 | TF |  |
| GSK3B | FGFR2 | ALDH9A1 |  |

**Table S5.** **Enriched GO items about immune system process of the predicted targets.**

| **GOID** | **GOTerm** | **Associated Genes Found** |
| --- | --- | --- |
| GO:0006957 | complement activation, alternative pathway | [AKR1C1, C3, C5, C8A, C8G, CFB, CFD] |
| GO:0002905 | regulation of mature B cell apoptotic process | [ADA, MIF, S100A8, S100A9] |
| GO:0002901 | mature B cell apoptotic process | [ADA, MIF, S100A8, S100A9] |
| GO:0002906 | negative regulation of mature B cell apoptotic process | [ADA, MIF, S100A8, S100A9] |

**Table S6.** **Enriched GO items about molecular function of the predicted targets.**

| **GOID** | **GOTerm** | **Associated Genes Found** |
| --- | --- | --- |
| GO:0002020 | protease binding | [ACR, CASP3, CEL, DPP4, ELANE, FN1, INS, ITGAV, KIT, LCN2, SERPINA1, SERPINA5, SERPINC1, TP53] |
| GO:0003707 | steroid hormone receptor activity | [AKR1B1, AR, ESR1, ESR2, NR1I2, NR1I3, NR3C1, NR3C2, PGR, PPARA, PPARG, RARA, RARB, RXRA, VDR] |
| GO:0004033 | aldo-keto reductase (NADP) activity | [AKR1B1, AKR1C1, AKR1C2, AKR1C3, AKR1C4, AKR7A2, AKR7A3, ALDH3A1, DHRS4, MIOX] |
| GO:0004175 | endopeptidase activity | [ACE, ACE2, ACR, ADAM17, ADAM33, BACE1, C2, C3, CASP1, CASP3, CASP7, CEL, CFB, CFD, CMA1, CTSD, CTSG, CTSL, CTSS, DPP4, ECE1, ELANE, F10, F11, F2, F5, F7, GZMB, KLK3, LPA, LTF, MASP2, MME, MMP1, MMP2, MMP9, PCSK1, PIP, PLAU, PLG, REN] |
| GO:0004252 | serine-type endopeptidase activity | [ACR, C2, C3, CEL, CFB, CFD, CMA1, CTSD, CTSG, CTSL, CTSS, DPP4, ELANE, F10, F11, F2, F5, F7, GZMB, KLK3, LPA, LTF, MASP2, MMP1, MMP2, MMP9, PCSK1, PLAU, PLG] |
| GO:0004553 | hydrolase activity, hydrolyzing O-glycosyl compounds | [AMY1A, AMY2A, CHI3L1, CHIA, CHIT1, GBA, GM2A, HEXA, HEXB, HYAL1, LYZ, MAN1A1, MAN1B1, MAN2B1] |
| GO:0004601 | peroxidase activity | [CAT, GPX3, GSTP1, GSTZ1, HBA1, HBB, IYD, MPO, PTGS1, PTGS2] |
| GO:0004672 | protein kinase activity | [ACVR2B, AKT1, AURKA, CCND1, CCNT1, CDK2, CSF2RB, CSNK2A1, DAPK1, EGF, EGFR, EPHA2, ERBB2, FGFR2, GSK3B, HSP90AA1, IGF1R, IGF2R, IL5, INSR, KDR, KIT, MAP2K1, MAPK1, MAPK14, MAPK8, MAPK9, NME2, PIK3CG, PIM1, PLK1, PRKACA, PRKCB, RNASE3, SRC, TRPM7] |
| GO:0004879 | RNA polymerase II transcription factor activity, ligand-activated sequence-specific DNA binding | [AKR1B1, AR, ESR1, ESR2, NR1I2, NR1I3, PPARA, PPARG, RARA, RARB, RXRA, VDR] |
| GO:0005501 | retinoid binding | [ALDH1A2, C8G, IGF2R, PTGDS, RARA, RBP1, RBP2, RBP4, RBP5, SERPINA5] |
| GO:0005504 | fatty acid binding | [ADH5, ALB, FABP1, FABP2, FABP3, FABP4, FABP5, NME2, PMP2, PPARG, PTGDS, S100A8, S100A9] |
| GO:0005506 | iron ion binding | [ALOX12, ALOX15, CYP2A13, CYP2C8, CYP2C9, CYP2E1, CYP7A1, HBA1, HBB, LCN2, LTF, MIOX, NOS1, NOS3, PTGS1, TF] |
| GO:0008201 | heparin binding | [ANG, APP, BMP7, CEL, CTSG, ELANE, F11, FGFR2, FN1, LPA, LTF, MPO, PGF, SELP, SERPINA5, SERPINC1, THBS1, THBS2, VEGFA] |
| GO:0008235 | metalloexopeptidase activity | [ACE, ACE2, CPA1, CPA4, CPB1, CPB2, CPO, CPOX, FOLH1, LTA4H] |
| GO:0008236 | serine-type peptidase activity | [ACE, ACR, C2, C3, CEL, CFB, CFD, CMA1, CTSD, CTSG, CTSL, CTSS, DPP4, DPP6, ELANE, F10, F11, F2, F5, F7, GZMB, KLK3, LPA, LTF, MASP2, MMP1, MMP2, MMP9, PCSK1, PLAU, PLG] |
| GO:0008237 | metallopeptidase activity | [ACE, ACE2, ADAM17, ADAM33, CEL, CPA1, CPA4, CPB1, CPB2, CPO, CPOX, ECE1, FOLH1, LTA4H, MME, MMP1, MMP2, MMP9] |
| GO:0008238 | exopeptidase activity | [ACE, ACE2, BACE1, CEL, CPA1, CPA4, CPB1, CPB2, CPO, CPOX, DPP4, DPP6, F11, FOLH1, LTA4H, MME] |
| GO:0008270 | zinc ion binding | [ACE, ACE2, ACR, ADA, ADAM33, ADH1B, ADH5, ADH6, AKR1B1, AR, BHMT, CA2, CPA1, CPA4, CPB1, CPB2, CPO, CPOX, CREBBP, DNMT1, ESR1, ESR2, GLO1, GRIN2A, LTA4H, MAN2B1, MME, MMP1, MMP2, MMP9, NR1I2, NR1I3, NR3C1, NR3C2, PARP1, PGR, PPARA, PPARG, PRKCB, PTGR2, PTPN1, QPCT, RARA, RARB, RELA, RTN4IP1, RXRA, S100A12, S100A8, S100A9, S100B, SIRT3, TP53, TRIM21, VDR] |
| GO:0016301 | kinase activity | [ACVR2B, ADK, AKT1, AURKA, CCND1, CCNT1, CDK2, CSF2RB, CSNK2A1, DAPK1, DCK, EGF, EGFR, EPHA2, ERBB2, FGFR2, GALK1, GALK2, GCK, GSK3B, HK2, HSP90AA1, IGF1R, IGF2R, IL5, INSR, KDR, KIT, KITLG, MAP2K1, MAPK1, MAPK14, MAPK8, MAPK9, NAGK, NME2, NME3, NME4, PCK1, PCK2, PIK3CG, PIM1, PKLR, PLAU, PLK1, PRKACA, PRKCB, PTPN11, RBKS, RFK, RNASE3, SRC, TRPM7, UCK2] |
| GO:0016616 | oxidoreductase activity, acting on the CH-OH group of donors, NAD or NADP as acceptor | [ADH1B, ADH5, ADH6, AKR1B1, AKR1C1, AKR1C2, AKR1C3, AKR1C4, AKR7A2, AKR7A3, ALDH3A1, DHRS4, G6PD, HMGCR, HSD11B1, HSD17B1, IDH1, IDH2, IMPDH1, IMPDH2, LDHA, LDHB, LDHC, MDH1, MDH2, ME1, MIOX, PTGR2, UEVLD] |
| GO:0016773 | phosphotransferase activity, alcohol group as acceptor | [ACVR2B, ADK, AKT1, AURKA, CCND1, CCNT1, CDK2, CSF2RB, CSNK2A1, DAPK1, EGF, EGFR, EPHA2, ERBB2, FGFR2, GALK1, GALK2, GCK, GSK3B, HK2, HSP90AA1, IGF1R, IGF2R, IL5, INSR, KDR, KIT, KITLG, MAP2K1, MAPK1, MAPK14, MAPK8, MAPK9, NAGK, NME2, PIK3CG, PIM1, PKLR, PLK1, PRKACA, PRKCB, PTPN11, RBKS, RFK, RNASE3, SRC, TRPM7] |
| GO:0019199 | transmembrane receptor protein kinase activity | [ACVR2B, EGFR, EPHA2, ERBB2, FGFR2, IGF1R, IGF2R, INSR, KDR, KIT] |
| GO:0019902 | phosphatase binding | [AKT1, CDH2, CTNNB1, EGFR, EIF4EBP1, ERBB2, GRIN3A, HMGCR, HSP90AA1, MAPK1, MAPK14, PPARA, PPARG, PTPN1, TP53] |
| GO:0019903 | protein phosphatase binding | [AKT1, CDH2, CTNNB1, EGFR, EIF4EBP1, ERBB2, GRIN3A, HMGCR, HSP90AA1, MAPK14, PPARG, PTPN1, TP53] |
| GO:0020037 | heme binding | [CAT, CYP2A13, CYP2C8, CYP2C9, CYP2E1, CYP7A1, HBA1, HBB, HEBP1, HMOX1, MB, MPO, NOS1, NOS2, NOS3, PTGS1, PTGS2, SRC] |
| GO:0031406 | carboxylic acid binding | [ADH5, AKR1C1, AKR1C2, ALB, DDAH1, DDC, DHFR, FABP1, FABP2, FABP3, FABP4, FABP5, GLUL, GRIN1, GRIN3A, GRIN3B, GRM7, IGF2R, NME2, NOS1, NOS2, NOS3, PCK1, PLA2G1B, PMP2, PPARG, PTGDS, PYGL, RARA, S100A8, S100A9, SELE, SELP, SERPINA5] |
| GO:0033293 | monocarboxylic acid binding | [ADH5, AKR1C1, AKR1C2, ALB, FABP1, FABP2, FABP3, FABP4, FABP5, IGF2R, NME2, PLA2G1B, PMP2, PPARG, PTGDS, PYGL, RARA, S100A8, S100A9, SERPINA5] |
| GO:0042803 | protein homodimerization activity | [ACHE, ADH5, ANG, ANXA1, AOC3, APOA2, BCL2A1, CASQ2, CAT, CD2, CEL, CPOX, DCK, DPP4, DPYD, ECE1, FGFR2, G6PD, GALE, GRIK2, GSTZ1, HEXB, HLA-G, HMGCR, HMGCS1, HMOX1, HSP90AA1, IDH1, IL6ST, IMPA1, IMPA2, KIT, LCN2, MAOB, MB, NOS2, NPR3, NUDT5, ODC1, PGF, PTGS2, PYGL, RELA, S100B, TOP2A, VEGFA] |
| GO:0046872 | metal ion binding | [ACE, ACE2, ACO1, ACR, ACVR2B, ADA, ADAM17, ADAM33, ADH1B, ADH5, ADH6, ADK, AKR1B1, ALB, ALOX12, ALOX15, AMY1A, AMY2A, ANG, ANXA1, AOC3, APCS, APEX1, APP, AR, ARF1, B3GAT1, BCKDHA, BHMT, C2, CA14, CA2, CANX, CASQ2, CAT, CD209, CDH2, CDK2, CLCNKA, CLEC4M, CP, CPA1, CPA4, CPB1, CPB2, CPO, CPOX, CPSF3, CREBBP, CYP2A13, CYP2C8, CYP2C9, CYP2E1, CYP7A1, DDAH1, DNMT1, DPYD, ECE1, EGF, ENO1, ESR1, ESR2, EXTL2, F10, F2, F5, F7, FDPS, FGA, FGG, FOLH1, GALNT1, GGPS1, GLO1, GLUL, GRIN1, GRIN2A, GRM7, GYG1, HAGH, HBA1, HBB, HMOX1, IDH1, IDH2, IMPA1, IMPA2, IMPDH1, IMPDH2, ITGAV, ITGB2, KIT, LCN2, LTA4H, LTF, MAN1A1, MAN1B1, MAN2B1, MASP2, MB, ME1, MIOX, MME, MMP1, MMP2, MMP9, MPO, NGLY1, NME2, NME3, NME4, NOS1, NOS2, NOS3, NOTCH1, NQO2, NR1I2, NR1I3, NR3C1, NR3C2, NT5M, NUDT5, NUDT9, PARP1, PCK1, PCK2, PDE5A, PDF, PGR, PIM1, PKLR, PLA2G10, PLA2G1B, PLA2G2A, PMM1, PNLIP, PNLIPRP1, PPARA, PPARG, PPP5C, PRKCB, PTGR2, PTGS1, PTGS2, PTPN1, QPCT, RARA, RARB, RBKS, RELA, RFK, RHO, RNASE3, RTN4IP1, RXRA, S100A12, S100A8, S100A9, S100B, SIRT3, SOD2, STEAP3, TF, TGM2, THBS1, THBS2, THTPA, TOP2A, TP53, TRIM21, TRPM7, VDR] |
| GO:0046914 | transition metal ion binding | [ACE, ACE2, ACR, ADA, ADAM33, ADH1B, ADH5, ADH6, AKR1B1, ALB, ALOX12, ALOX15, ANG, AOC3, APP, AR, BHMT, CA2, CP, CPA1, CPA4, CPB1, CPB2, CPO, CPOX, CREBBP, CYP2A13, CYP2C8, CYP2C9, CYP2E1, CYP7A1, DNMT1, ESR1, ESR2, F5, GALNT1, GLO1, GLUL, GRIN2A, HBA1, HBB, IMPA1, LCN2, LTA4H, LTF, MAN2B1, ME1, MIOX, MME, MMP1, MMP2, MMP9, NOS1, NOS3, NR1I2, NR1I3, NR3C1, NR3C2, PARP1, PCK1, PGR, PIM1, PPARA, PPARG, PRKCB, PTGR2, PTGS1, PTGS2, PTPN1, QPCT, RARA, RARB, RELA, RTN4IP1, RXRA, S100A12, S100A8, S100A9, S100B, SIRT3, SOD2, TF, TP53, TRIM21, VDR] |
| GO:0050661 | NADP binding | [CAT, DHFR, DPYD, G6PD, GSR, HMGCR, IDH1, ME1, NOS1, NOS2, NOS3] |
| GO:0070011 | peptidase activity, acting on L-amino acid peptides | [ACE, ACE2, ACR, ADAM17, ADAM33, BACE1, C2, C3, CASP1, CASP3, CASP7, CEL, CFB, CFD, CMA1, CPA1, CPA4, CPB1, CPB2, CPO, CPOX, CTSD, CTSG, CTSL, CTSS, DPP4, DPP6, ECE1, ELANE, F10, F11, F2, F5, F7, FOLH1, GZMB, KLK3, LPA, LTA4H, LTF, MASP2, MME, MMP1, MMP2, MMP9, PCSK1, PIP, PLAU, PLG, REN] |

**Table S7.** **Enriched KEGG pathway** **of the predicted targets.**

| **GOID** | **GOTerm** | **Associated Genes Found** |
| --- | --- | --- |
| GO:0000010 | Glycolysis / Gluconeogenesis | [ADH1B, ADH5, ADH6, ALDH2, ALDH3A1, ALDH9A1, ENO1, GALM, GCK, HK2, LDHA, LDHB, LDHC, PCK1, PCK2, PKLR, TPI1] |
| GO:0000270 | Cysteine and methionine metabolism | [AHCY, AMD1, BCAT1, BCAT2, BHMT, DNMT1, LDHA, LDHB, LDHC, MDH1, MDH2] |
| GO:0000350 | Tyrosine metabolism | [ADH1B, ADH5, ADH6, ALDH3A1, AOC3, DDC, GSTZ1, MAOA, MAOB, MIF] |
| GO:0000590 | Arachidonic acid metabolism | [AKR1C3, ALOX12, ALOX15, CYP2C8, CYP2C9, CYP2E1, GPX3, LTA4H, PLA2G10, PLA2G1B, PLA2G2A, PTGDS, PTGS1, PTGS2] |
| GO:0000620 | Pyruvate metabolism | [ACOT12, ALDH2, ALDH9A1, GLO1, HAGH, LDHA, LDHB, LDHC, MDH1, MDH2, ME1, PCK1, PCK2, PKLR] |
| GO:0000980 | Metabolism of xenobiotics by cytochrome P450 | [ADH1B, ADH5, ADH6, AKR1C1, AKR7A2, AKR7A3, ALDH3A1, CYP2A13, CYP2C9, CYP2E1, GSTP1, GSTT2B, HSD11B1, SULT2A1] |
| GO:0003320 | PPAR signaling pathway | [ACADM, APOA2, CYP7A1, FABP1, FABP2, FABP3, FABP4, FABP5, FABP7, ME1, MMP1, PCK1, PCK2, PPARA, PPARG, RXRA] |
| GO:0004014 | Ras signaling pathway | [AKT1, ARF6, EFNA1, EGF, EGFR, EPHA2, FGFR2, GRIN1, GRIN2A, IGF1R, INS, INSR, KDR, KIT, KITLG, MAP2K1, MAPK1, MAPK8, MAPK9, PGF, PIK3CG, PLA2G10, PLA2G1B, PLA2G2A, PRKACA, PRKCB, PTPN11, RAC1, RAC3, RELA, RHOA, VEGFA] |
| GO:0004015 | Rap1 signaling pathway | [ADORA2A, AKT1, CRKL, CTNNB1, EFNA1, EGF, EGFR, EPHA2, FGFR2, GRIN1, GRIN2A, IGF1R, INS, INSR, ITGB2, KDR, KIT, KITLG, MAP2K1, MAPK1, MAPK14, PGF, PIK3CG, PRKCB, RAC1, RAC3, RHOA, SRC, THBS1, VEGFA] |
| GO:0004066 | HIF-1 signaling pathway | [AKT1, CREBBP, EGF, EGFR, EIF4EBP1, ENO1, ERBB2, HK2, HMOX1, IGF1R, INS, INSR, LDHA, MAP2K1, MAPK1, NOS2, NOS3, PIK3CG, PRKCB, RELA, TF, VEGFA] |
| GO:0004068 | FoxO signaling pathway | [AKT1, CAT, CCND1, CDK2, CREBBP, EGF, EGFR, IGF1R, IL10, INS, INSR, MAP2K1, MAPK1, MAPK14, MAPK8, MAPK9, PCK1, PCK2, PIK3CG, PLK1, SOD2] |
| GO:0004370 | VEGF signaling pathway | [AKT1, KDR, MAP2K1, MAPK1, MAPK14, NOS3, PIK3CG, PRKCB, PTGS2, RAC1, RAC3, SRC, VEGFA] |
| GO:0004510 | Focal adhesion | [AKT1, CCND1, CRKL, CTNNB1, EGF, EGFR, ERBB2, FN1, GSK3B, IGF1R, ITGAV, KDR, MAP2K1, MAPK1, MAPK8, MAPK9, PGF, PIK3CG, PRKCB, RAC1, RAC3, RHOA, SRC, THBS1, THBS2, VEGFA] |
| GO:0004520 | Adherens junction | [CREBBP, CSNK2A1, CTNNB1, EGFR, ERBB2, IGF1R, INSR, MAPK1, PTPN1, PTPRM, RAC1, RAC3, RHOA, SRC] |
| GO:0004610 | Complement and coagulation cascades | [C2, C3, C5, C8A, C8G, CFB, CFD, CPB2, F10, F11, F2, F5, F7, FGA, FGG, ITGB2, MASP2, PLAU, PLG, SERPINA1, SERPINA5, SERPINC1, SERPING1] |
| GO:0004917 | Prolactin signaling pathway | [AKT1, CCND1, CGA, ESR1, ESR2, GCK, GSK3B, INS, MAP2K1, MAPK1, MAPK14, MAPK8, MAPK9, PIK3CG, RELA, SRC] |
| GO:0004933 | AGE-RAGE signaling pathway in diabetic complications | [AKT1, CASP3, CCND1, FN1, IL1B, MAPK1, MAPK14, MAPK8, MAPK9, MMP2, NFATC1, NOS3, PIK3CG, PIM1, PRKCB, RAC1, RELA, SELE, VEGFA] |
| GO:0005133 | Pertussis | [C2, C3, C5, CASP1, CASP3, CASP7, IL10, IL1B, ITGB2, MAPK1, MAPK14, MAPK8, MAPK9, NOS2, RELA, RHOA, SERPING1] |
| GO:0005150 | Staphylococcus aureus infection | [C2, C3, C5, CFB, CFD, FCAR, FCGR2A, FGG, HLA-DRB1, IL10, ITGB2, MASP2, PLG, SELP] |
| GO:0005200 | Pathways in cancer | [AKT1, AR, CASP3, CCND1, CDK2, CREBBP, CRKL, CTNNB1, DAPK1, DCC, EGF, EGFR, ERBB2, FGFR2, FN1, FZD8, GSK3B, GSTP1, HSP90AA1, IGF1R, ITGAV, KIT, KITLG, KLK3, MAP2K1, MAPK1, MAPK8, MAPK9, MMP1, MMP2, MMP9, NOS2, PGF, PIK3CG, PPARG, PRKACA, PRKCB, PTGS2, RAC1, RAC3, RARA, RARB, RELA, RHOA, RXRA, TP53, VEGFA] |
| GO:0005204 | Chemical carcinogenesis | [ADH1B, ADH5, ADH6, AKR1C2, ALDH3A1, CYP2A13, CYP2C8, CYP2C9, CYP2E1, GSTP1, GSTT2B, HSD11B1, PTGS2, SULT1A1, SULT2A1] |
| GO:0005205 | Proteoglycans in cancer | [AKT1, CASP3, CCND1, CTNNB1, CTSL, EGFR, ERBB2, ESR1, FN1, FZD8, IGF1R, ITGAV, KDR, MAP2K1, MAPK1, MAPK14, MMP2, MMP9, PIK3CG, PLAU, PRKACA, PRKCB, PTPN11, RAC1, RHOA, SRC, THBS1, TP53, VEGFA] |
| GO:0005210 | Colorectal cancer | [AKT1, CASP3, CCND1, CTNNB1, DCC, GSK3B, MAP2K1, MAPK1, MAPK8, MAPK9, PIK3CG, RAC1, RAC3, RHOA, TP53] |
| GO:0005212 | Pancreatic cancer | [AKT1, CCND1, EGF, EGFR, ERBB2, MAP2K1, MAPK1, MAPK8, MAPK9, PIK3CG, RAC1, RAC3, RELA, TP53, VEGFA] |
| GO:0005215 | Prostate cancer | [AKT1, AR, CCND1, CDK2, CREBBP, CTNNB1, EGF, EGFR, ERBB2, FGFR2, GSK3B, GSTP1, HSP90AA1, IGF1R, INS, KLK3, MAP2K1, MAPK1, PIK3CG, RELA, TP53] |
| GO:0005219 | Bladder cancer | [CCND1, DAPK1, EGF, EGFR, ERBB2, MAP2K1, MAPK1, MMP1, MMP2, MMP9, SRC, THBS1, TP53, VEGFA] |
| GO:0005230 | Central carbon metabolism in cancer | [AKT1, EGFR, ERBB2, FGFR2, G6PD, GCK, HK2, IDH1, KIT, LDHA, MAP2K1, MAPK1, PIK3CG, SIRT3, TP53] |
